# A scalable variational method for estimating the latent infection-rate field of an outbreak

**DOI:** 10.1371/journal.pone.0350090

**Published:** 2026-05-29

**Authors:** Wyatt H. Bridgman, Cosmin Safta, Jaideep Ray

**Affiliations:** Sandia National Laboratories, Livermore, California‌‌, United States of America; Villanova University, UNITED STATES OF AMERICA

## Abstract

In this paper, we explore whether the infection-rate of a disease can serve as a robust monitoring variable in epidemiological surveillance algorithms. The infection-rate is dependent on population mixing patterns that do not vary erratically day-to-day; in contrast, daily case-counts used in contemporary surveillance algorithms are corrupted by reporting errors. The technical challenge lies in estimating the latent infection-rate from case-counts. Here we devise a Bayesian method to estimate the infection-rate across multiple adjoining areal units, and then use it, via an anomaly detector, to discern a change in epidemiological dynamics. We extend an existing model for estimating the infection-rate in an areal unit by incorporating a Markov random field model, so that we may estimate infection-rates across multiple areal units, while preserving spatial correlations observed in the epidemiological dynamics. To carry out the high-dimensional Bayesian inverse problem, we develop an implementation of mean-field variational inference specific to the infection model and integrate it with the random field model to incorporate correlations across counties. The method is tested on estimating the COVID-19 infection-rates across all 33 counties in New Mexico using data from the summer of 2020, and then employing them to detect the arrival of the Fall 2020 COVID-19 wave. We perform the detection using a temporal algorithm that is applied county-by-county. We also show how the infection-rate field can be used to cluster counties with similar epidemiological dynamics.

## 1 Introduction

There have been many attempts to estimate the infection-rate of an outbreak [1–3], especially for the COVID-19 pandemic, including our own work [4–6]. In these studies, the infection-rate is modeled as a time-varying function, which is estimated by fitting a disease model to observed data, e.g., case-counts of symptomatic (diagnosed) individuals per day. The infection-rate is used to forecast the outbreak over a small time horizon, e.g., two weeks, and then compared with the data from that period. This ability to compare observed case-counts with model forecasts can be used to fashion an outbreak detector for disease surveillance – if the model forecasts and reported case-counts do not match, it could indicate a change in the epidemiological dynamics, either due to a change in human mixing patterns, e.g., due to lock-downs [6] or due to a new variant of the pathogen [4]. A shortcoming of these studies is that they do not contain any spatial information on the variation of the infection-rate, which impairs their usefulness in disease surveillance – public health policy is determined globally (e.g., for a nation) and then adapted and applied locally (e.g., in a county). Being able to estimate an infection-rate *field*, defined over multiple areal units (e.g., counties), can thus be very helpful. However, the task is challenging, as the problem now requires one to use epidemiological data, e.g., case-counts, collected from each areal unit, which could have a small population. Such data, gathered from small populations, tends to be contaminated with high-variance noise (reporting errors) and has to be compensated for by imposing the spatial patterns extant in epidemiological dynamics caused by mixing of humans between neighboring areal units. In addition, the estimation procedure will need to smoothen the noisy case-count data.

In this paper, we develop a method to estimate the latent infection-rate field of an outbreak. Though general, we demonstrate it on COVID-19 data from the 33 counties of New Mexico (NM). It consists of two parts (1) an epidemiological model that employs a parametrized spatio-temporal model of the infection-rate field and (2) a scalable, if approximate, mean-field variational inference (MFVI) algorithm that allows us to estimate the parameters of the infection-rate field, the noise and the spatial variation over all of NM. The epidemiological model, which will be derived in § 3.1, is an extension of an older model [4,6] with a Gaussian Markov Random Field (GMRF) model to represent spatial correlations. This model has been tested for a small problem consisting of three adjoining NM counties using adaptive Markov chain Monte Carlo (AMCMC [7]) as the estimation procedure. AMCMC does not make any assumptions regarding the nature of the posterior distributions of the parameters being estimated. This small study is available as a preprint [8] and has been published [9].

**Fig 1:**
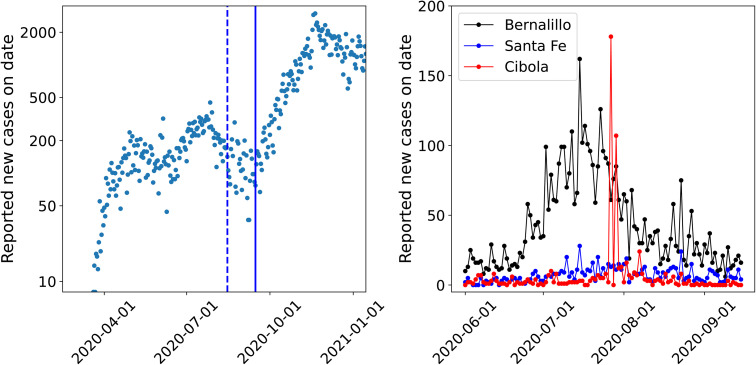
Left: The three COVID-19 waves in NM in 2020. We see the Spring wave, ending around June 1^st^, followed by the Summer wave and the Fall wave that started around September 15^th^. Our aim is to estimate the infection-rate *field* during the Summer wave and detect the Fall 2020 wave. The solid vertical line denotes September 15^th^ and the dashed one is placed at August 15^th^. Right: Plots of case-counts from three counties. While Bernalillo and Santa Fe show reasonably smooth variation, the two large peaks from Cibola are clearly erroneous.

AMCMC will not scale to all 33 NM counties, necessitating the use of MFVI. In MFVI, one imputes a parametrized posterior distribution for the infection-rate field; in our case, we model the posterior distribution of (transformations of) our parameters as independent Gaussians of unknown means and standard deviations. These means and standard deviations are estimated by minimizing an objective function using an iterative, gradient-based algorithm. We will test our method by reconstructing the time-dependent infection-rate field over all 33 counties of NM, with case-count data of diverse qualities, collected during 2020. During that year, the COVID-19 outbreak in NM consisted of 3 waves, of which the “Fall 2020” wave that started around September 15^th^ was the largest; these are plotted in Fig 1 (left). We will use the observational data from the “Summer 2020” wave, spanning June 1^st^ to September 15^th^, to estimate the infection-rate field, and fashion a detector for the Fall 2020 wave with it. Briefly, we will use the infection-rate field, as it was computed on September 15^th^ to forecast two weeks ahead, and compare with the data. They will not agree, as the infection-rate field has no information about the Fall 2020 wave; being able to isolate this disagreement quickly is the figure of merit for the detector. This detection was performed for Bernalillo, Santa Fe and Valencia counties in Ref. [8,9] and the Fall 2020 wave was detected wi^th^in a week of September 15^th^.

The research questions of this study are:

- How can MFVI be used to solve the estimation problem efficiently, given that many of the parameters are non-negative? Can transformations be used to render this a problem of *unconstrained* optimization?
- How does the approximate posterior distribution employed in MFVI compare against the “true” one computed using AMCMC? By necessity, this comparison will be limited to the three counties included in it.
- The spatial correlations embedded in the field estimation problem allow an areal unit to “borrow information” from its neighbors and compensate for poor-quality data. How robust is this correlation, i.e., when it fails due to poor quality data, does it do so catastrophically (i.e., the estimation process stops) or gracefully, with a non-informative (or erroneous) estimate?
- How does the approximate infection-rate field estimated by MFVI affect the accuracy of the outbreak detector? Is MFVI sufficiently accurate to be useful, despite its inherent approximations? Further, does the infection-rate field, estimated over all NM counties, reveal anomalous spatial structures during the start of the Fall 2020 wave?

We state the modeling premises employed for this paper:

- Our aim is to estimate the latent infection-rate field of the outbreak; it is *not* just to forecast accurately.
- We do not use a proper conditional auto-regressive space-time model (pCAR-ST) to model the infection-rate across multiple areal units. This is intentional. It is partly motivated by the studies of Lawson and collaborators [10–12] who found that a model similar to a pCAR-ST worked well in some cases and in others [10], it did not. Instead, we extend an older model of ours [6] with a pCAR, as it had performed well for single areal units.
- Unlike a pCAR-ST model, our model allows the imposition of smoothing time-scales (in the form of priors) that are exogenous to the data. In our case, they arise from the incubation period of the disease and the time profile of the outbreak. The necessity for doing so (which arises from low quality data from an areal unit) will be discussed in § 5.

The paper has three main contributions. Our first contribution is the determination of the degree to which the predictive skill of the disease model forecast is affected by the use of an imputed and approximate posterior distribution; we find that it is not much vis-à-vis the AMCMC solution. Our second contribution is the discovery that despite the approximations inherent in MFVI, the infection-rate field so estimated retains sufficient information on the outbreak to detect epidemiological anomalies. Note that we will not attempt to make a proper outbreak detector in this paper; that is left to future work. Our third contribution is of a numerical nature. Our disease model includes a convolutional integral making it difficult (and expensive) to compute the gradient of the objective function, analytically or by finite differences. Our innovations lie in how the convolution and the gradient are computed numerically via quadrature and the reparametrization of the objective function that allows us to use an unconstrained optimization method, despite the need to preserve non-negativity of parameters being estimated.

The paper is structured as follows. In § 2, we review relevant literature. In § 3 we formulate the inverse problem and the MFVI adaptation, and results are presented in § 4. Outbreak detection is explored § 5. We conclude in § 6.

## 2 Literature review

In this section, we review some of the literature that undergird our spatiotemporal epidemiological model, as well as work on variation inference algorithms that will be used to scale our model to high-dimensional inverse problems involved in estimating infection-rate fields.

### 2.1 Spatial modeling in epidemiology‌‌

Epidemiological dynamics show spatial auto-correlation because of human mixing, as well as the dependence of some outbreaks on socioeconomic and demographic covariates which do not change erratically in space or time. The COVID-19 pandemic was observed and recorded with fine spatiotemporal granularity, and the data has been subjected to much spatiotemporal modeling. Many such studies found that the spread of the disease was mediated mostly by human mixing, rather than by socioeconomic factors; Huang *et al.* [13] found it to be so in Hubei province in China. Geng *et al.* [14] analyzed US data and found that spatial patterns’ length-scales ranged from the county-level to the nation; similar results were found by Schuler *et al.* [15] for Germany. McMahon *et al.* [16] analyzed data at the county-level and found that the correlation length-scales for spatial variability changed during the course of the pandemic; further, the correlation in epidemiological dynamics between urban centers were stronger than elsewhere. Indika *et al.* [17] analyzed data from the counties of Virginia and found that spatial auto-correlation of case-counts, as quantified by Moran test statistics, were impacted by, and linked to, executive orders at the state level. Thus, COVID-19 has presented us with much evidence of spatial auto-correlation in epidemiological dynamics.

The incorporation of spatial auto-correlation in the modeling and estimation of infection-rate fields is rare; however, it has been extensively used in disease mapping. In disease maps, one develops a field, called *relative risk* *r*_*k*_, that is used to adjust an expected value of morbidity *e*_*k*_ to the locally observed case-counts $y_{k}^{\left(obs\right)}$ in an areal unit (e.g., county) *k*, often via a Poisson or Negative Binomial link, i.e., $y_{k}^{\left(obs\right)}\sim Poisson(r_{k}e_{k})$. The expected value *e*_*k*_ is usually obtained from a regional average. The relative risk *r*_*k*_ is modeled using covariates of disease activity, e.g., socioeconomic conditions $\log(r_{k})=z_{k}\cdot \beta +\phi_{k}$, where ***z***_*k*_ are co-variate risk factors for areal unit *k*, β are regression weights and $\phi_{k}$ captures auto-correlated random effects in space using a random field model. The simplest random field model is iCAR (intrinsic Conditional AutoRegressive [18]), a specific type of Gaussian Markov Random Field (GMRF). Thus


$$\begin{array}{c} \phi =\{\phi_{k}\}\sim N\left(0,\{\tau^{2}Q{\}}^{-1}\right),\;Q=diag(W1)-W, \end{array}$$

where *W* is the adjacency matrix of the areal units (i.e., $w_{ij}=1$ if areal units *i* and *j* share a boundary). The object of estimation from data is $\tau^{2}$. The precision matrix *Q* tends to be sparse. Another common model is the Besag-York-Mollie (BYM) model [19] which decomposes $\phi_{k}$ as $\phi =\phi^{1}+\phi^{2},\phi^{1}\sim N(0,\{\tau^{2}Q{\}}^{-1})$ and $\phi^{2}\sim N(0,\sigma^{2}I)$. An adaptation of the BYM is used in this paper. More details on spatial models is available in our preprint [4,6], where we also test the spatial model employed in the current paper on a smaller problem with three counties.

The method described in this paper is a spatial extension of a method in our previous paper [6] which was designed to provide short-term forecasts of an outbreak, conditional on daily case-counts data. One starts with a parameterized model of the time-varying (latent) infection-rate (*f*_*inf*_) and convolves it with the cumulative distribution of the incubation period (*F*_*inc*_) to obtain an integral equation for the for the case-counts (very similar to Eq. 2). *f*_*inf*_ and *F*_*inc*_ are fashioned as Gamma functions/distributions. This model was used to forecast COVID-19 case-counts in a number of countries and US states, each of them treated as a single areal unit. This method was then extended to multi-wave epidemics [4] and used to forecast outbreaks in California, New Mexico and Florida after multiple waves. These were high-dimensional estimation problems, as each wave had its own infection-rate parameters, and were solved using AMCMC. The spatial model for forecasting and detecting the “Fall 2020” wave on multiple areal units was developed in Refs. [8,9]. One of the innovation in Refs. [8,9] was the method used to model the spatial correlations in disease dynamics., driven by case-count data; it was found that, in NM, this was limited to nearest-neighbors among NM counties. Note that this will not hold true for other states with differently-sized areal units, population densities and mixing patterns. Refs. [8,9] also contain details on the GMRF model and how it was selected; the same GMRF is used in this paper. This infection-rate estimation was performed using AMCMC; due to its limited scalability, the estimation was limited to three adjoining counties in Ref. [8]. The estimation and the forecasts were very accurate and detection of the Fall 2020 wave was feasible within about a week’s worth of data. It was this study that motivated us to extend our work to all 33 NM counties by developing a scalable, if approximate, MFVI method that is described in this paper. It adapts the method in Refs. [8,9], via transforms, to be amenable for MVFI and unconstrained optimization.

The work done by Lawson and collaborators [10–12] is the closest to our own. Fundamentally we extend an older model of ours [4,6], meant for a single areal unit, to encompass multiple areal units. Each areal unit contains its own parametrized infection-rate representation, but are “stitched” together using a BYM model. The parameters are estimated by solving an inverse problem, conditioned on case-count data from areal units. Our model also includes a model for the incubation period. In contrast, Lawson and co-workers model case-counts directly; the clearest exposition of the model in in Ref. [10], and it has been used with COVID-19 data from South Carolina [20] and the UK [21]. Lawson and co-workers, much like us, have used the disagreement between calibrated model forecasts and data as signs of epidemiological anomalies and devised metrics such as the Surveillance Kullback-Liebler [22] (SKL) and Surveillance Conditional Predictive ordinate [23] (SCPO) to detect them.

### 2.2 Variational inference

Inverse problems for model calibration often require the approximation of intractable probability densities arising from Bayesian inference. A standard approach based on sampling is Markov Chain Monte Carlo (MCMC) [24] but suffers from scalability issues due to slow convergence rates for high-dimensional and/or multi-modal distributions [25,26]. Variational inference (VI) [27] provides an alternative to sampling techniques where approximate inference is recast as seeking a member of a family of approximating densities which minimizes a discrepancy measure such as KL-divergence. Originally developed for probabilistic graphical models [28] where some degree of analytical tractability is maintained, it has more recently been extended to many-parameter models, such as those seen in deep learning, through gradient-based iterative schemes adapted to a probabilistic setting [29–31]. These techniques are often termed Stochastic Variational Inference (SVI) and can exploit the automatic differentiation available in large ML models. They offer significantly improved scalability over MCMC while potentially sacrificing some approximation quality depending on the set of approximating distributions used. VI has seen successful applications to a number high-dimensional inverse problems in areas including medical classification [32,33] and segmentation [34,35], computer vision and image processing [36], natural language processing [37,38], and physics-based models [39,40].

In addition to this broad range of application spaces, VI has more recently been adopted in a growing number of epidemiological modeling challenges. Neural ODEs have been extended to a Bayesian setting where they can be calibrated with VI and applied to state space epidemiological models [41]. Model selection and dynamic causal modeling based on evolving real-world time series using VI have been applied to COVID-19 outbreaks [42,43] to provide online forecasting tools. Dynamical system inference for spatio-temporal modeling of infectious diseases using ODE and PDE formulations has been carried out at the state scale and combined with Bayesian neural networks [2]. Model calibration with VI has also been explored using alternatives to standard state-space models such as graph-coupled Hidden Markov Models [44]. Hassan *et al.* [45] developed a modified SEIR mathematical model to forecast COVID-19 trends. This study combined deterministic modeling with a Continuous-Time Markov Chain (CTMC) approach to calculate outbreak probability, emphasizing the critical need for coordinated public health measures. Kamrujjaman *et al.* [46] developed a modified SEIR mathematical model for COVID-19 that combines exposed and asymptomatic populations into a single compartment and includes the effects of panic, tension, and anxiety on mortality rates across susceptible, exposed, and infected classes. They identified disease transmission rates and panic-related mortality as the most sensitive parameters. In addition to predictive model calibration, generative probabilistic modeling using VI-based approximations of distributions has also been explored to generate mission information about disease spread [47].

## 3 Formulation

Here we propose an epidemiological model to forecast infection-rates across adjacent geographical regions and use these forecasts to detect emergent outbreaks. The geographical regions / areal units can have data of very diverse qualities. Fig 1 (right) shows case-count data from three counties of NM during the Summer wave. Cibola clearly shows anomalies in the data with two peaks in late-July 2020 that do not admit any epidemiological explanations. The model that we construct will need to be robust to such erroneous data, which can be achieved via spatial and temporal smoothing. The “usual” method for smoothing such a field would be via a space-time proper Conditional AutoRegressive (pCAR-ST; [48]) model. Such an approach, using a pCAR model and a one-step-ahead-in-time (as in AR(1)) was explored by Lawson and collaborators [10,12] who found that the method was not much better than a purely temporal model [10], though later results with COVID-19 data showed otherwise [20,21]. In order to avoid this uncertainty, we extend an older (temporal) method of ours [4,6] with a GMRF model to enable the estimation of an infection-rate field and perform multi-region outbreak detection. This older model has been tested on COVID-19 data from NM [4,6].

### 3.1 Epidemiological model

The epidemiological model is defined by a spatio-temporally varying infection-rate model and an incubation model given by


$$\begin{array}{c} f_{inf}(t;t_{0}^{r},k^{r},\theta^{r})=\frac{(\theta^{r}{)}^{-k}(t-t_{0}^{r}{)}^{k-1}}{\Gamma (k^{r})}\exp\left(-\frac{t-t_{0}^{r}}{\theta^{r}}\right) \end{array}$$

$$\begin{array}{c} F_{inc}(t;\mu ,\sigma )=\frac{1}{2}erfc\left(-\frac{\log t-\mu }{\sigma \sqrt{2}}\right) \end{array}$$ (1)

where the infection-rate *f*_*inf*_ is a Gamma distribution with shape and scale parameters *k*^*r*^ and $\theta^{r}$, respectively. Note that 1 ≤ *r* ≤ *R* indexes the spatial region. $F_{inc}(t;\mu ,\sigma )$ is the cumulative distribution of the incubation period for COVID-19, taken from Lauer *et al*. [49]; further details to the temporal model are in [4,6]. The parameter $t_{0}^{r}$ represents the start of the outbreak and will be inferred along with the infection-rate parameters. The number of people that turn symptomatic over the time interval [*t*_*i*−1_, *t*_*i*_] is given by


$$\begin{array}{c} y_{r}(i)=y_{r}(t_{i};t_{0}^{r},N^{r},k^{r},\theta^{r}) \end{array}$$

$$\begin{array}{c} =N_{r}\int_{t_{0}^{r}}^{t_{i}}f_{inf}\left(\tau -t_{0}^{r};k^{r},\theta^{r}\right)\left[F_{inc}(t_{i}-\tau ;\mu ,\sigma )-F_{inc}(t_{i-1}-\tau ;\mu ,\sigma )\right]d\;\tau \end{array}$$ (2)

so that $𝐲(i)={𝐲}_{i}={\left[y_{1}(i)\cdots y_{R}(i)\right]}^{T}$ and *i* represents the time-dependence of the predictions. Here, *N*^*r*^ is the fourth and final region-dependent parameter and represents the total number of people infected during the entire epidemic wave in spatial region *r* normalized by the population of region *r*.

In the discussion above, *f*_*inf*_ was chosen to be a Gamma distribution, primarily for simplicity of parameterization. For more complex temporal behavior of the latent infection-rate, more involved parameterizations may be required, e.g., the parameterization for multi-wave outbreaks, as described in Ref. [4].

The noisy model predictions are defined as


$$\begin{array}{c} {𝐲}_{i}^{\left(o\right)}={𝐲}_{i}^{\left(p\right)}+\epsilon_{i}=ℳ(t_{i};𝐦)+\epsilon_{i},\;\epsilon_{i}\sim 𝒩(0,\Sigma_{i}). \end{array}$$ (3)

Here $𝐦=vec\left({𝐦}_{r}\right)$, where ${𝐦}_{r}^{T}=(t_{0}^{r},N^{r},k^{r},\theta^{r})$, are the region-specific model parameters for r=1,…,R and ℳ(:) is an epidemiological / disease model that uses $f_{inf}(:)$. In addition, ${𝐲}_{i}^{\left(o\right)}$ is the vector of observed case-counts on day *i* for all *R* regions, ${𝐲}_{i}^{\left(p\right)}$ is the vector of *R* case-count predictions for the same day using the model ℳ and $\epsilon_{i}$ is the “noise” observed case-counts on day *i* that cannot be explained by ℳ (mostly reporting errors). To account for spatial correlations and heteroscedastic noise seen in case-counts, the noise is assumed to be composed of two terms $\epsilon_{i}=\epsilon_{i,1}+\epsilon_{i,2}$ where the first is given by a Gaussian Markov Random Field (GMRF) model while the second represents temporally-varying, independent Gaussian noise. Letting 𝒟 and Θ represent the data and parameters, respectively, the likelihood then takes the form


$$\begin{array}{c} p(𝒟\;|\;\Theta )=\prod_{i=1}^{N_{d}}\frac{1}{(2\pi {)}^{N_{r}/2}det\Sigma_{i}^{1/2}}\exp\left(-\frac{1}{2}({𝐲}_{i}^{\left(o\right)}-{𝐲}_{i}^{\left(p\right)})\Sigma_{i}^{-1}({𝐲}_{i}^{\left(o\right)}-{𝐲}_{i}^{\left(p\right)}{)}^{T}\right) \end{array}$$ (4)

where $\Sigma_{i}$ is given by


$$\begin{array}{c} \Sigma_{i}=\tau_{\Phi }P^{-1}+diag{\left(\sigma_{a}+\sigma_{m}{𝐲}_{i}^{\left(p\right)}\right)}^{2}. \end{array}$$ (5)

The first term $P=\left[D-\lambda_{\Phi }𝐖\right]$ in Eq. (5) forms the precision matrix of a GMRF component of the noise where the strength of correlations induced by adjacent regions is governed by $\lambda_{\Phi }$. The relative topology of regions is encoded by **W**, the county adjacency matrix, defined as


$$\begin{array}{c} w_{kk}=0\;\;\text{and}\;\;w_{kl}=\left\{ \begin{array}{cc} 1 & \text{if regions}kandlareadjacent\}\\ 0 & \text{otherwise} \end{array}\right. \end{array}$$ (6)

Here, $D=diag\{g_{1},g_{2},\ldots ,g_{R}\}$ where *g*_*i*_ is the number of regions adjacent to region *i*. Note that this formulation assumes that the *R* spatial regions are self-contained with negligible population mixing with the region around them. The derivation of the adjacency matrix **W** and its subsequent inclusion in $\Sigma_{i}$ is presented in detail in Refs. [8,9], and we reproduce a summary here. In Refs. [8,9], we plotted the population-normalized case-counts, summed over 90-day periods, for all the NM counties and noticed a spatial correlation between counties along the populated Rio Grande valley; furthermore, the spatial correlation persisted over a moving 90-day period. We examined the spatial correlation between a county and its neighborhood, where a neighborhood was defined as the set of counties that shared a border with the county in question, i.e., “one-hop” neighbors. A two-sided Moran’s *I*-statistic test revealed that the spatial correlation was supported by the evidence. We also considered a neighborhood of one- and two-hop neighbors and the same test found no evidence for it. This allowed us to design an adjacency matrix consisting of immediate neighbors only and formulate a simple precision matrix for the GMRF model for the correlated errors (defined as the difference between observed case-counts on a day *i* in a given county and model predictions $Y_{i}^{\left(p\right)}$). We also add a heteroscedastic reporting error to the diagonal of the GMRF’s covariance matrix $\Sigma_{i}$ equal to ${\left(\sigma_{a}+\sigma_{m}Y_{i}^{\left(p\right)}\right)}^{2}$. For the purposes of this paper, we have modeled NM in isolation, i.e., the adjacency matrix **W** does not contain terms that link counties along the NM border to the neighboring counties in adjoining states. This is an approximate boundary condition of no population mixing outside NM which is necessary to limit the scope of the problem, else **W** would grow unbounded. Note that the counties along the NM boundary are large, sparsely-populated, mountainous or desert regions with little mixing with the population outside, making feasible this approximation.

The second term captures prediction-dependent, uncorrelated noise with additive and multiplicative components governed by $\sigma_{a}$ and $\sigma_{b}$, respectively. The relative contribution between the correlated GMRF noise and the uncorrelated noise is controlled by $\tau_{\Phi }$. Full details of the development of *P* can be found in Refs. [8,9]. Using Bayes’ rule, one can obtain an expression for the posterior density of Θ, the parameters to be estimated from data, as:


$$\begin{array}{c} p(\Theta |vec({𝐲}^{\left(o\right)}))\propto p(vec({𝐲}^{\left(o\right)})|\Theta )\pi (\Theta ), \end{array}$$

where Θ is defined in § 3.2 and $vec({𝐲}^{\left(o\right)})=\{{𝐲}_{i}^{\left(o\right)}\}$. For low-dimensional problems, the density $p(\Theta |vec({𝐲}^{\left(o\right)}))$ can be obtained by sampling directly. This was performed in Ref. [8,9] for three adjoining NM counties (Bernalillo, Santa Fe and Valencia) using AMCMC [7] as a test of the correctness of the formulation. Using data from the Summer wave, we developed PDFs of ${𝐦}_{r},r=1\ldots 3$, $\tau_{\Phi },\lambda_{\Phi },\sigma_{a},$ and $\sigma_{m}$ and forecast the case-counts for two weeks beyond September 15^th^, 2020, in order to detect the arrival of the Fall 2020 wave (the forecasts beyond September 15 and the observed case-counts would disagree). We could detect the arrival of the Fall wave with a week’s worth of observations. We also tested the method using data gathered till August 15^th^ to check the detector’s susceptibility to false positives. However, due to the lack of scalability of AMCMC (in terms of parameters being estimated and parallel computing), we cannot use it to estimate the infection-rate field in all 33 counties of NM and therefore take recourse to a mean-field variational inference techniques that, though approximate, will scale to the 126 parameters that the NM-wide estimation will require.

### 3.2 Statistical inference

The set of parameters Θ defining the likelihood in Eq. (4) is given by


$$\begin{array}{c} \Theta =vec\left(\theta_{i}\right)=vec\left([\begin{array}{cccc} {𝐦}_{1} & \cdots & {𝐦}_{R} & \eta \end{array}]\right) \end{array}$$ (7)

where $\eta =(\tau_{\Phi },\lambda_{\Phi },\sigma_{a},\sigma_{m})$ are the global noise parameters. Inference consists of forming the posterior distribution p(Θ | 𝒟)=p(𝒟 | Θ)p(Θ)/p(𝒟) over uncertain parameters Θ. As the posterior is intractable, we instead look to approximate it using VI. Hence, the following sections describe how VI is formulated carried out to approximate the posterior p(Θ | 𝒟) for the outbreak model as well as how the prior p(Θ) is defined to regularize the inverse problem.

#### 3.2.1 Variational inference.

We will compare the Bayesian posterior sampled with AMCMC with posterior models obtained using MFVI which recasts approximate inference as an optimization problem. In particular, as the exact posterior is intractable, we consider a family of approximating densities $ℱ=\{q(\Theta ;\phi )\;|\;\phi \in \Phi \subseteq {\mathbb{R}}^{d}\}$ and seek to find a density $q(\Theta ;\phi^{*})$ that minimizes the KL-divergence with respect to the posterior


$$\begin{array}{c} \phi^{*}=argmin\;D_{KL}\left(q(\Theta ;\phi )\;\|\;p(\Theta \;|\;𝒟)\right) \end{array}$$ (8)

This can be re-expressed as minimizing the objective function ℒ(ϕ) based on the evidence lower bound (ELBO) [30]


$$\begin{array}{c} ℒ(\phi )=-ℍ\left[q(\Theta ;\phi )\right]-{𝔼}_{q(\Theta ;\phi )}\left[\log p(𝒟\;|\;\Theta )+\log p(\Theta )\right] \end{array}$$ (9)

where the first term in Eq. (9) is the entropy of the surrogate posterior and the second, data-dependent term is an expectation with respect to the surrogate posterior that reflects both the expected data-fit and the prior. Here we take ℱ to be the set of mean-field Gaussian distributions, i.e.,


$$\begin{array}{c} q\left(\Theta ;\;\phi \right)=\prod_{i=1}^{d}q_{i}\left(\theta_{i};\;\mu_{i},\sigma_{i}\right) \end{array}$$ (10)

where $q_{i}(\theta_{i};\mu_{i},\sigma_{i})=𝒩(\theta_{i};\mu_{i},\sigma_{i})$, ϕ=(μ,σ). We arrive at an optimization problem over 2*d* parameters where *d* is the number of parameters defining the epidemiological model ℳ. To carry out the above minimization problem, we aim to use a gradient-based iterative scheme as the expectation in Eq. (9) cannot be evaluated explicitly due to the nonlinearity of the forward model. Furthermore, ℒ(ϕ) is potentially a non-convex objective. Note that the gradient and expectation operators do not commute, i.e.,


$$\begin{array}{c} \nabla_{\phi }{𝔼}_{q\left(\Theta ;\phi \right)}\left[\log p\left(𝒟\;|\;\Theta \right)p(\Theta )\right]\neq {𝔼}_{q\left(\Theta ;\phi \right)}\left[\nabla_{\phi }\log p\left(𝒟\;|\;\Theta \right)p\left(\Theta \right)\right] \end{array}$$

so some care has to be taken to arrive at a Monte Carlo estimator for the gradient $\nabla_{\phi }ℒ(\phi )$. Two widely used approaches are: (a) the score function estimator, described in § A.1 (in the Appendix), which forms the basis of black-box VI and requires only evaluations of the log-likelihood, and (b) the reparametrization approach which requires gradients of the log-likelihood. The score function estimator typically displays much larger variance as seen in Kucukelbir *et al.* [50] where two orders of magnitude more samples were needed to arrive at the same variance as a reparametrization estimator. A similar trend was confirmed for the outbreak problem (Fig 9) suggesting that the reparametrization approach would lead to superior scalability. Reparametrization proceeds by expressing Θ as a differentiable transformation Θ=t(ϵ,ϕ) of a ϕ-independent random variable ϵ~q(ϵ) such that Θ(ϵ,ϕ)~q(Θ,ϕ). This allows the gradient to be expressed as


$$\begin{array}{c} \nabla_{\phi }ℒ\left(\phi \right)=-\nabla_{\phi }ℍ\left[q\left(\Theta ;\phi \right)\right]-{𝔼}_{q\left(\epsilon \right)}\left[\nabla_{\phi }\log p\left(𝒟\;|\;\Theta (\epsilon ,\phi )\right)+\nabla_{\phi }\log p\left(\Theta \left(\epsilon ,\phi \right)\right)\right] \end{array}$$ (11)

where gradients of the entropy term in Eq. (11) are available analytically for the Gaussian surrogate posterior and the second term can now be approximated with Monte Carlo given a method to compute the required gradients. For many machine learning models, automatic differentiation can be exploited to calculate the gradient of the log-likelihood with respect to parameters Θ. Here, the objective function involves the log of the likelihood (Eq. (4)) where derivatives of matrix inverses and determinants with respect to parameters are required to compute the gradient. Gradients such as these are not available using most automatic differentiation libraries. Instead, matrix calculus and quadrature were used to compute the derivatives of the log-likelihood with respect to model predictions ${𝐲}_{i}^{\left(p\right)}$ and to approximate the derivatives of the model predictions with respect to parameters, respectively. For details, see § A (in the Appendix).

Note that some of the parameters comprising Θ are required to satisfy constraints for the noise and epidemiological models to be well-defined. For example, noise parameters $\tau_{\Phi }$, $\sigma_{a}$, and $\sigma_{m}$ as well as model parameters *N*^*r*^, *k*^*r*^ and $\theta^{r}$ for r=1,…,R should be positive while $\lambda_{\Phi }$ should satisfy $0\leq \lambda_{\Phi }<1$. Sampling from the mean-field Gaussian (Eq. (10)) during the Monte Carlo estimation of the gradient (Eq. (11)) may result in violations of these constraints. To maintain the required properties without resorting to constrained optimization, we express a constrained parameter $\theta_{i}$ as an invertible, differentiable transformation $\theta_{i}=f_{i}({\hat{\theta }}_{i})$ of an unconstrained ${\hat{\theta }}_{i}$. Hence, the distribution governing $\theta_{i}$ is the push-forward density of $𝒩({\hat{\theta }}_{i};\mu_{i},\sigma_{i})$ through *f*_*i*_, i.e., the components of the mean-field surrogate posterior (Eq. (10)) have modified probability densities


$$\begin{array}{c} q_{i}(\theta_{i};\mu_{i},\sigma_{i})=𝒩({\hat{\theta }}_{i};\mu_{i},\sigma_{i})|f_{i}^{\prime }({\hat{\theta }}_{i}){|}^{-1};\;1\leq i\leq d \end{array}$$ (12)

where ${\hat{\theta }}_{i}=f_{i}^{-1}(\theta_{i})$. This results in mean-field approximation where some of the factors are Gaussian and others non-Gaussian. Each factor is still defined by a $\mu_{i}$ and $\sigma_{i}$ parameter. The transformations are listed in Table 2.

The initial motivation for using Gaussian distributions can be seen in Fig 5 where the posterior approximations from AMCMC display negligible skew and kurtosis suggesting a variational distribution with vanishing higher-order moments is sufficient to capture the behavior of interest. Second, as the goal was to produce a method for efficient high-dimensional inference, scalability and analytical tractability had to be balanced with accuracy tradeoffs. Note that MFVI also has a tendency to underestimate the uncertainty / variance in the estimated parameters [51] and in § 4, we will check if this poses a limitation on the usefulness of the approximate solution, especially in the predictive skill of model ℳ and therefore the detection of the start of outbreaks.

#### 3.2.2 Prior distribution.

The COVID-19 case count data exhibits significant noise due to inaccurate case counting reported by hospitals. Furthermore, counties with small populations exhibit sparse data in the sense that not many positive daily case counts were reported. Hence, we expect the inverse problem to be ill-posed and require regularization in the form of a prior p(Θ) over the parameters.

Because of push-forward formulation described by Eq. (12), a number of the parameters are already constrained by transformations $\theta_{i}=f_{i}({\hat{\theta }}_{i})$. In particular, the parameters $N^{r},k^{r},\theta^{r}$ for r=1,…,R and each of the noise parameters comprising η are all constrained by transformations to take on values in some restricted interval. For example, $\lambda_{\Phi }$ is constrained to lie within [0,1−ϵ], for some ϵ>0 so that Eq. (5) defines a valid covariance matrix, i.e., it remains symmetric, positive definite. The parameters $t_{0}^{r}$ are the only unconstrained variables. Hence, we take Gaussian priors over $t_{0}^{r}$ that incorporate diffuse assumptions about when it is reasonable for a wave to occur.

#### 3.2.3 Posterior predictive tests.

The mean-field variational inference (MFVI) described in § 3.2.1 results in a multivariate Gaussian posterior distribution (Eq. (12)) that then needs to be verified against data 𝒟. To do so, we take samples $\Theta_{j}\sim q\left(\Theta ;\phi \right)$, and using Eq. (3), generate predictions $Y_{j}=\{{𝐲}_{i}^{\left(p\right)}+\epsilon_{i}{\}}_{j},j=1\ldots J$. For this paper, *J* = 100. The time-series ***Y***_*j*_ result in a “fantail” of predictions for each areal unit *r* which should statistically reproduce 𝒟, e.g., the inter-quartile range of the samples ***Y***_*j*_ should bound 50% of the observations and the 5^th^ and 95^th^ percentiles should bound 90% of the individual data points in 𝒟. This test is called a posterior predictive test (PPT) which we will use extensively in § 4. In addition, we define a score to summarize the agreement of ***Y***_*j*_ with 𝒟. Predictive distributions constituted out of $\{{𝐲}_{i}^{\left(p\right)}{\}}_{j},j=1\ldots J$ samples are called “push-forward“ (PF) predictions.

Define $y_{r,i}^{\left(pred\right)}(\Theta )=y_{r}(i;\;{𝐦}_{r})+\epsilon_{r}(\eta )$ to be the model prediction for region *r* for day *i* by combining Eq. (3) and Eq. (7). Let $y_{r,i,j}=y_{r,i}^{\left(pred\right)}(\Theta_{j})$ be the prediction corresponding to a sample $\Theta_{j}$ drawn from the posterior. Let $y_{r,i}^{\left(obs\right)}$ be the corresponding observation (from 𝒟). Let $G_{r,i}(y^{\left(pred\right)};\Theta )$ be the cumulative distribution function (CDF) for the model predictions arising from the posterior distribution q(Θ;ϕ). The Continuous Ranked Predictive Score (CRPS [52]) is defined as


$$\begin{array}{c} c_{r,i}=\int {\left(G_{r,i}(\upsilon )-1_{\upsilon >y_{r,i}^{\left(obs\right)}}\right)}^{2}d\upsilon \;\text{and}\;\text{CRPS}=\frac{\sum_{i}^{N_{d}}c_{r,i}}{N_{d}}. \end{array}$$ (13)

The CRPS has units of case-counts and will be larger for areal units with larger total case-counts $T_{r}=\sum_{i}y_{r,i}^{\left(obs\right)}$, and we will use the ratio $\rho_{r}=\text{CRPS}/T_{r}$ to compare across areal units in § 4. In practice, the empirical CDF computed using *J* samples of ***Y***_*j*_ is used to approximate $G_{r,i}(y^{\left(pred\right)};\Theta )$. This score function has been used in judge the quality of the model to capture the spread in the data used for calibration. [6,53,54] We use the implementation in the R Statistical Software [55] (R version 4.3.2 (2023-10-31)) package verification [56], specifically the function crpsDecomposition(), to compute the CRPS.

## 4 Results

In this section, the calibration of the outbreak model, using the formulation presented in § 3, is studied across several cases. The COVID-19 pandemic arrived in NM in March 2020; Fig 1 (left) plots the detected cases in NM over 2020. We see three clear “waves“ - the Spring wave, the Summer wave which spanned June 1^st^ to September 15^th^, and the Fall wave that arrived after that. Unless specified otherwise, data from the Summer wave (i.e., June 1, 2020 to September 15, 2020) is used to perform the estimation and the estimated infection-rate is used to forecast two weeks ahead, into the Fall wave. The COVID-19 dataset of case-counts that we use covers the duration from 2020-01-22 to 2022-05-13, and consists of daily (new) case-counts of COVID-19 from each of the 33 counties of NM; the data is available online. [57,58]. The sudden change in epidemiological dynamics around September 15^th^ implies that that the infection-rate profile from the Summer wave will not be able to forecast the Fall wave; therefore any disagreement between the observed data and forecasts is a sign of the arrival of the Fall wave.

First, we consider three populated counties that are adjacent to each other and display a large number of cases. These counties present less noise and clearer trends resulting in a more well-posed inversion task. **W** is limited to these three counties, implying that there is no population mixing outside them. This is an approximation and will likely affect the estimated infection-rate’s accuracy. The aim of the study is to compare the effect of calibrating multiple areal units jointly using MFVI, as well as to compare against the calibration performed using AMCMC [8,9]. Next, the coupled outbreak model is calibrated across all 33 New Mexico counties. This represents a more challenging task given the dimensionality of the problem as well as a multitude of counties displaying sparse and noisy case-counts. We then evaluate the final posterior approximation using PPT runs. The convergence of the MVFI procedure for both the outbreak model and noise parameters is investigated and discussed last. We next discuss anomaly detection using the MFVI-calibrated outbreak model is carried out using COVID-19 spread-rates across all 33 counties in New Mexico using data from the summer of 2020 to detect the arrival of the Fall 2020 COVID-19 wave. In this study, all the counties of NM are included in **W**, implying that there is negligible cross-border mixing of the population with the adjoining states. This is approximately true, as the border counties of NM are remote, sparsely-populated deserts and mountains.

When estimating the infection-rate field in the 3-counties, we infer 3×4+4=16 parameters – 4 parameters $\left(t_{0}^{r},N_{r},k^{r},\theta^{r}\right)$ for each county and 4 noise parameters $\left(\tau_{\phi },\lambda ,\sigma_{a},\sigma_{m}\right)$. For all of NM, with its 33 counties, the dimensionality of the inverse problem is 33×4+4=136 independent parameters. This is too large for AMCMC and was the motivation for developing MFVI.

The MFVI procedure follows § 3 where the stochastic gradient descent iteration is carried out using the Adaptive Moment Estimation (ADAM) algorithm [60]. MFVI is well-known to display mode-seeking behavior due properties of the KL-divergence. Hence, to facilitate the convergence of MFVI, we initialize the mean parameters for MFVI from a Maximum Likelihood Estimate (MLE) which is readily available using gradients of the log-likelihood. During calibration via stochastic gradient descent, $N_{s}=200$ samples were used in the Monte Carlo estimates of the ELBO gradient Eq. (11).

### 4.1 Three-county inversion

In this section we investigate the effect of using an approximate MFVI inversion by comparing against AMCMC solution [8,9]. We also check the effect of estimating the infection-rate across multiple areal units vis-à-vis independently.

#### 4.1.1 Joint versus independent calibrations.

**Fig 2:**
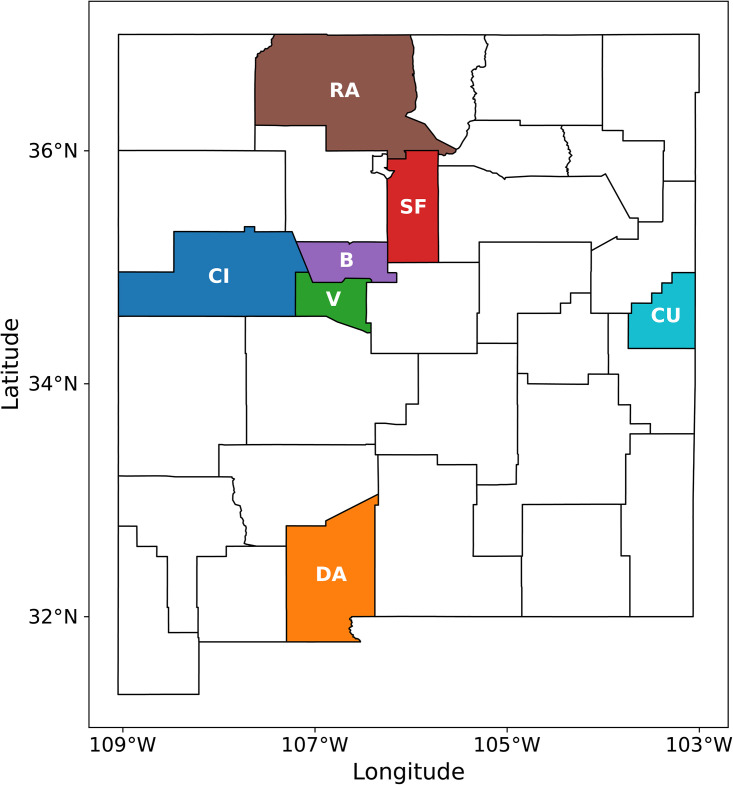
The counties of New Mexico. The shaded ones are where we will present results. Abbreviations: B = Bernalillo; CI = Cibola; CU = Curry; DA = Doña Ana; RA = Rio Arriba SF = Santa Fe and V = Valencia. The shapefiles of the counties were downloaded from the US Census Bureau website [59].

**Fig 3:**
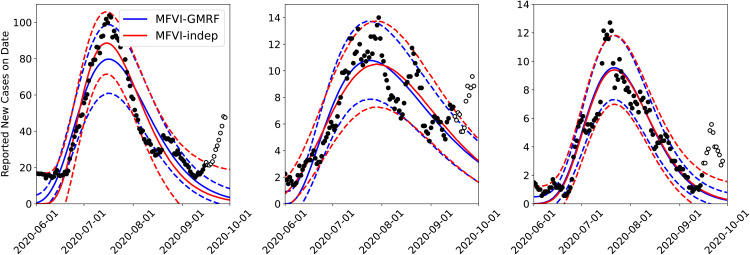
Comparison of the predictive distribution for the MFVI inversions of Bernalillo (left), Santa Fe (middle), and Valencia (right) done jointly using the *GMRF* model and *independently* for each county. The solid lines show median predictions and the dashed lines bound the 5^th^–95^th^ quantile interval. Case-count data was smoothed with a 7-day running average. The filled symbols are the data 𝒟 used in the inversion. The unfilled symbols are the observations beyond September 15^th^ and are used to compare with the two-week forecasts.

First, we perform an infection-rate estimation for three adjoining counties – Bernalillo, Santa Fe and Valencia – using MFVI independently and jointly using the spatial (GMRF) model (Eq. (5)); see Fig 2 for their positions. In Fig 3, the results of a PPT for all three counties are displayed in both the joint and independently calibrated cases. The case-count data were smoothed with a 7-day running average. The median prediction is plotted with the red line and the dashed lines denote the 5^th^ and 95^th^ percentiles. We see that most of the observations (filled symbols) are within these bounds. We also see the 2-week-ahead forecast beyond September 15th and the unfilled symbols showing the observed data from that period. The forecasts and the data do not match for any of the three counties, indicating a change in the epidemiological dynamics. This change is due to the arrival of the Fall 2020 wave of COVID-19 in NM, and the figure shows that the wave arrived approximately simultaneously in all three counties (see Fig 1 (left) for a clearer picture of the three COVID-19 waves encountered in 2020 in NM). Observe that the jointly calibrated predictive distribution displays less uncertainty in the predictions (i.e., small $\sigma_{m}$) than the independently calibrated version. This is likely due to a combination of two factors. The first is that incorporated spatial correlations between counties regularizes the calibration and results in more certainty about the true underlying model parameters. The second is that the uncorrelated MFVI approximation is known to underestimate uncertainty for highly correlated distributions. Note also that uncertainty is largest in the predictive distribution for Santa Fe consistent with the markedly noisier behavior of the case-counts for this county. The infection-rate profiles for these counties are in Fig 14 in the Appendix. There is not much difference between them indicating that the infection-rate parameter estimates in the two cases might be similar, whereas the estimate of the noise, which affects PPT results, vary between the two formulations due to the fact that the spatial noise model has to accommodate all the noise, across all the areal units, together. These findings echo what we observed when the same study was performed using AMCMC as the estimation procedure [8,9].

#### 4.1.2 AMCMC versus MFVI estimation.

**Fig 4:**
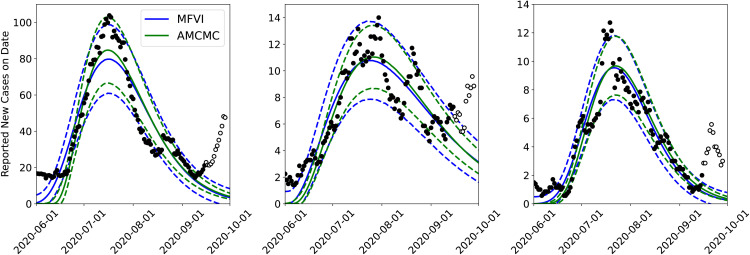
Comparison of the predictive distribution for the joint inversion of Bernalillo (left), Santa Fe (middle), and Valencia (right) done jointly using *MFVI* and *AMCMC.* The solid lines show median predictions and the dashed lines bound the 5^th^–95^th^ quantile interval. Case-count data (filled and unfilled circles) are the same as in Fig 3. The AMCMC results are taken from Refs. [8,9].

Next, we study the effect of using our approximate MFVI method versus the estimates computed using AMCMC [8,9]. In Fig 4 we plot the PPT results from the joint MFVI (top) and AMCMC (bottom) for the same three counties. Here we see that the MFVI estimates a larger $\sigma_{m}$ – the 5^th^ and 95^th^ percentile bounds (dashed lines) are far wider for MFVI results vis-à-vis AMCMC results below. By dint of having wider bounds, the MFVI estimate is also better at bounding the data used to compute the infection-rate *field* for the three counties. Again the arrival of the Fall 2020 wave is clearly discerned in the figure. The infection-rate profiles for these counties are in Fig 15 in the Appendix and there is not much difference between them.

**Fig 5:**
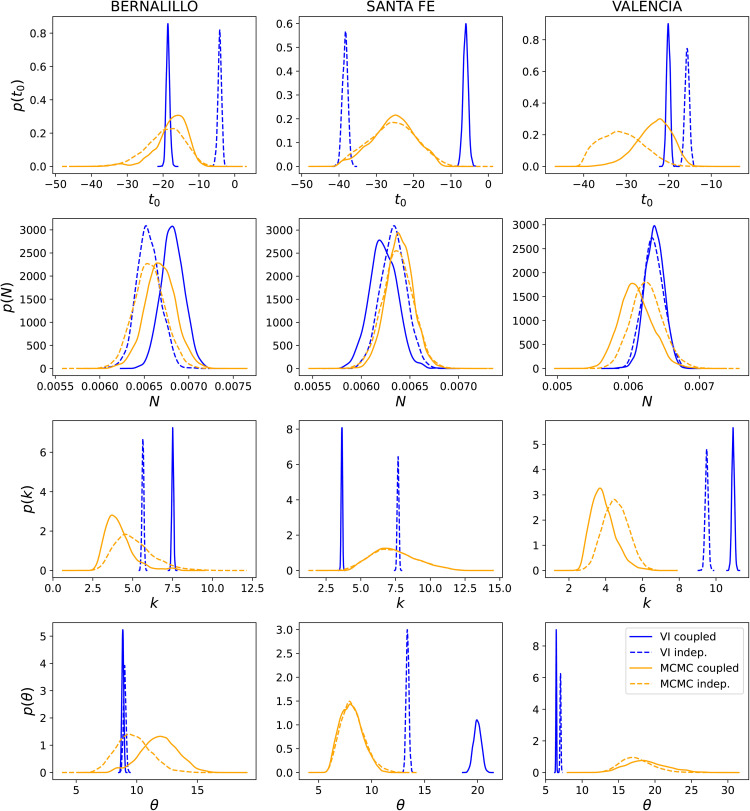
Comparison of the posterior over model parameters *t*_0_, *N*, *k*, and θ for 3-county inference of Bernalillo (left), Santa Fe (middle), and Valencia (right) using both MCMC (orange) and MFVI (blue). Both joint (solid lines) and independent calibration (dashed lines) are results are displayed. *t*_0_ values are negative as it is measured from June 10^th^, 2020, and *t*he PDFs imply that infections for the Summer wave started in late May. ’VI indep.’ in the legend implies an estimate obtained for each county independently using MFVI.

In Fig 5, we summarize the marginalized posteriors for the infection-rate parameters for Bernalillo, Santa Fe, and Valencia computed using MFVI and AMCMC, jointly and independently. $t_{0}^{r}$ assumes negative values as it is measured from June 10^th^, 2020; the PDFs peak around −20 (for AMCMC results) and imply that the infections for the Summer wave started in late May, about 20 days before June 10^th^. The MFVI results also agree approximately with this estimation. We see that apart from *N*_*r*_, the MFVI and AMCMC posteriors do not match. The MFVI posteriors are extremely narrow, providing a spurious degree of certainty in the estimates; this arises from the form of the posterior distribution – independent Gaussians – that we postulate in Eq. 10. In addition, as is clear from the AMCMC results, the “true” posteriors are not Gaussian. The marginalized posteriors computed via AMCMC, for joint and independent estimation, do match (sometimes very well, as in the case of Valencia), but they are wide apart for the MFVI, showing the effect of the Gaussian approximation. Yet the infection-rate profiles in Fig 14 (in the Appendix) do not show much of a difference, nor do the PPT results in Fig 3, leading us to conjecture that the influence of some of these parameter on the infection-rate may be muted. This can also be surmised from the marginal distributions computed from AMCMC – they are quite wide.

The difference in the posterior densities of the parameters (Fig 4, joint estimation only) deserves an explanation. They arise from the assumptions of independence inherent in MFVI. In reality, the posterior density is strongly correlated, and these can be computed using AMCMC (see Refs. [8,9]). The Gaussian assumption in MFVI also plays a role, but it is of lower consequence as the AMCMC posteriors are not excessively skewed, and a Gaussian is an acceptable approximation.

Finally, we summarize the predictive skill of the joint estimates computed using MFVI and AMCMC in Table 1 using CRPS. The CRPS, computed over data between June 1^st^ and September 15^th^, 2020, summarizes the agreement of the PPTs with observations for each of the counties. We see that the CRPS (a measure of the error between predictions and data) is about 10 cases per day, for Bernalillo (where case-counts peaked at about 100 cases/day; see Fig 3) and 2.5 cases a day for Valencia and Santa Fe (which peaked at about 12 cases a day). What is remarkable is the difference in the CRPS as computed using MFVI and AMCMC – it is less than a case-count per day. This small change in the predictive skill leads us to believe that the scalable, but approximate, MFVI approach might be sufficiently accurate to allow us to detect the arrival of the Fall 2020 wave in an automated manner.

**Table 1:** Predictive skill of PPTs generated using infection-rate estimates computed using different procedures. All estimations are performed using the joint formulation, using the spatial model. The PPTs are scored using CRPS. They have units of “case-counts”.

Procedure	Bernalillo	Santa Fe	Valencia
AMCMC, 3-county	11.3	2.65	1.76
MFVI, 3-county	10.75	2.54	1.70
MFVI, 33-county	10.7	2.35	1.47

Taken from Table 2 of Refs. [8,9].

Computed from Fig 3 (top).

*To summarize*, from Figs 3–5 it is clear that:

- Joint estimation performed with a GMRF model leads to more certain forecasts compared to independent estimation of infection-rates in areal units (i.e., counties).
- Marginalized posterior obtained from MFVI shows spuriously low levels of uncertainty compared to AMCMC.
- Despite the difference in the posterior densities computed using MFVI and AMCMC, the forecasts are not very sensitive to them; this is because the differences in the infection-rate are compensated by the reporting errors’ parameters $\sigma_{a}$ and $\sigma_{m}$. However, the forecasts performed using MFVI’s posterior densities are slightly more uncertain. Therefore anomaly detection with them will also be a little less accurate.

### 4.2 Joint inversion of all NM counties

**Fig 6:**
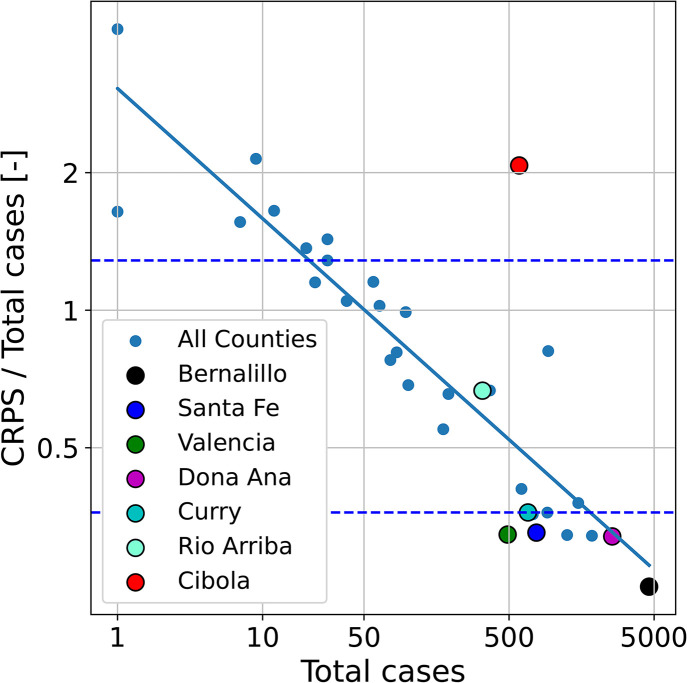
CRPS/*T* plotted as a function of total number of cases *T.* Both the axes are log-axes. We see, as expected, that the predictive skill of the disease model, post calibration, is better for counties with larger case-counts *T* where noise variance is low. The horizontal dashed lines are the first and third quartiles of ρ=CRPS/T.


Next, calibration of the full 33-county model using MFVI was carried out, followed by PPT runs which were then summarized using CRPS computed for each NM county. In Fig 6, we plot the CRPS normalized by the total number *T* of cases a function of *T*, for all counties. Data between June 1^st^ and September 15^th^ was used in the infection-rate estimation as well as the computation of CRPS is a measure of the “goodness of model fit” to data. We see that the CRPS, as a ratio of the total cases, decreases with increasing number of total cases, as the disease model fits larger outbreaks in counties like Bernalillo. Others, like Cibola, do not agree with model predictions, due to flaws in the data, as we will see later in Fig 12. The straight line fit to data has the form

$$\begin{array}{c} \log(\rho )=\log\left(\frac{\text{CRPS}}{T}\right)\approx 1.1-0.28\log(T), \end{array}$$

This equation shows that ρ scales (approximately) as the fourth root of the total number of cases, a slow reduction indeed.

**Fig 7:**
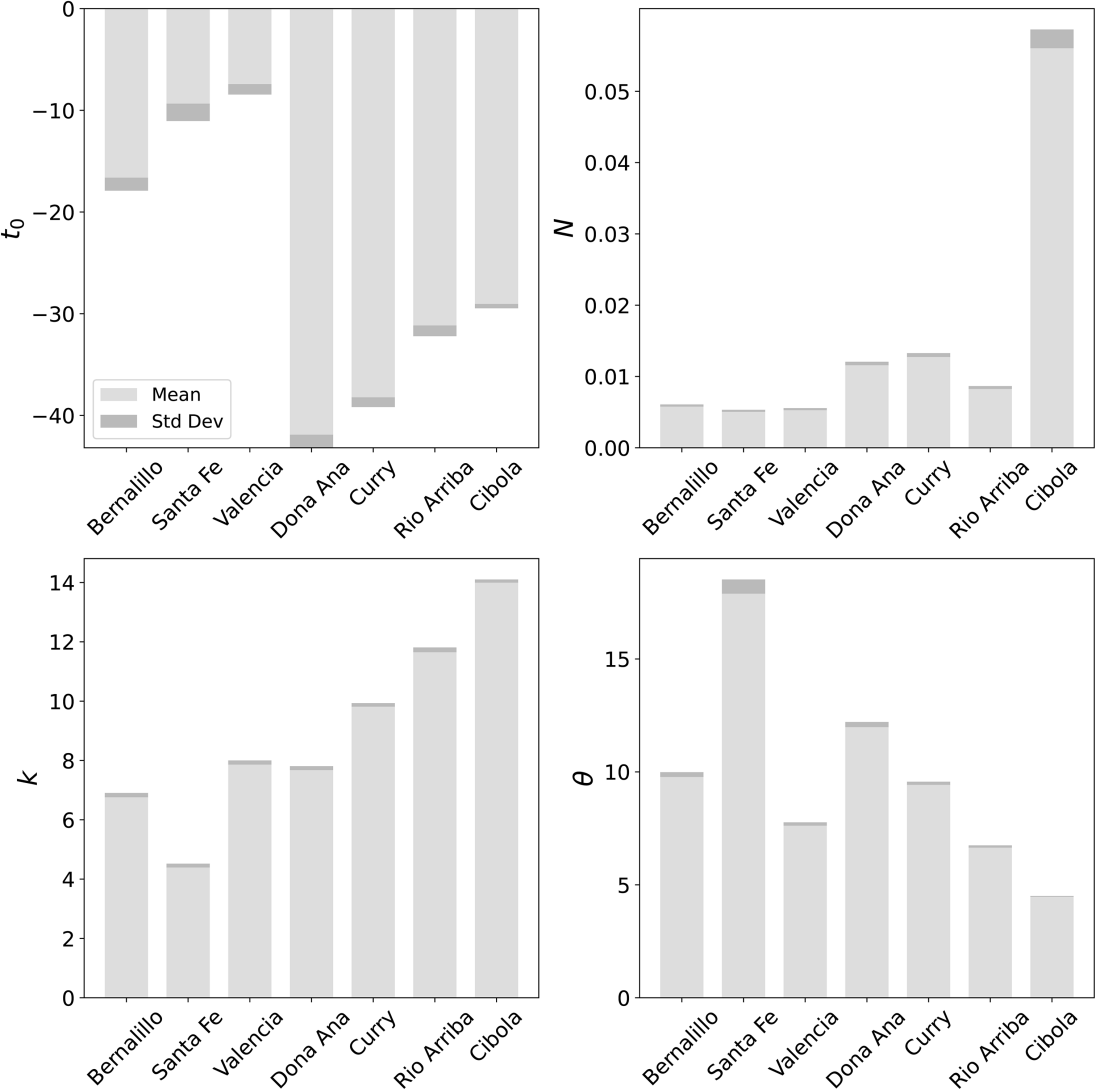
Means (light gray) and standard deviation (dark gray) of various infection-rate parameters, for select counties marked in Fig 6. Top left: The start time of the Summer wave t_0_. Top right: The total size of the Summer outbreak *N*. Bottom left: *k*, the shape parameter of the Gamma profile of the infection-rate. Bottom right: *θ*, the scale parameter of the Gamma profile.

The horizontal lines in Fig 6 show the first and third quartiles of ρ. The three counties studied in § 4.1, Bernalillo, Santa Fe and Valencia, are marked and fall in the lowest quartile, i.e., their data is good and the calibrated disease model is predictive. We will use the county of Doña Ana as another member of the “good” class, while Curry and Rio Arriba, which fall in the inter-quartile range of ρ, will serve as exemplars of the “middling” class of calibration. Cibola, which falls in the last quartile, will be an exemplar of the “bad” class of calibration. We now examine the quality of the inversion in each of these counties. In Fig 7 we plot the infection-rate parameters, as estimated from the Summer wave case-count data, for 7 counties marked in Fig 6. We see that the standard deviations are spuriously tiny, in line with what was observed in Fig 5; thus MFVI consistently underestimates the uncertainty in the parameter estimates. Further, this spuriously low uncertainty is pervasive – counties with high CRPS such as Cibola and Rio Arriba show much the same estimation uncertainties as counties such as Bernalillo and Santa Fe with CRPSs a factor of three smaller. Thus the uncertainty in the parameters’ estimates do not seem credible and we will omit them from further discussion. However, despite the quality of the case-count data, the inversion completes *stably* and provides plausible results. However, as Fig 6 shows, the infection-rate may not be estimated very accurately in some counties and this might hamper the task of detecting the Fall 2020 wave reliably.

**Fig 8:**
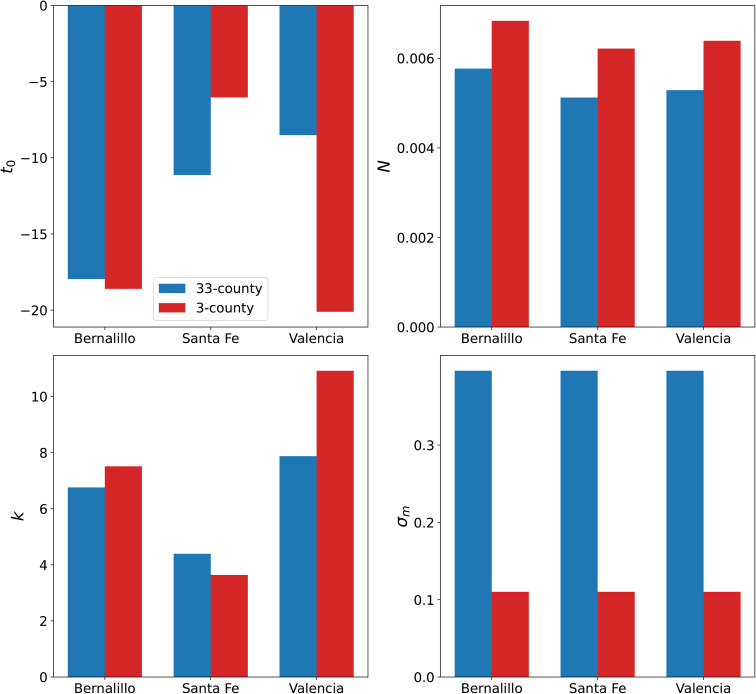
Select infection-rate and noise parameters, estimated jointly among three counties, compared with their counterparts from a 33-county inversion. The noise parameter $\sigma_{m}$ is markedly larger in the 33-county inversion.

In Fig 8 we compare some infection-rate parameters $\left(t_{0}^{r},N_{r},k^{r},\sigma_{m}\right)$ for Bernalillo, Santa Fe and Valencia, in the 3-county (as plotted in Fig 5) and 33-county inversions. MFVI was used for both the computations. We see that the parameters are not the same and there does not seem to be a clear trend, except that $\sigma_{m}$ increases when we include counties (some with poor quality data) in the estimation. This implies that the PPTs for the “good” counties will likely be wider than what could be achieved with AMCMC and this might have repercussions regarding detecting the Fall 2020 wave.

*To summarize*, the uncertainty in the estimated parameters, computed using MFVI, are not very credible. However, the MFVI inversion is stable, though the uncertainty in the PPTs will be larger because of an inflated $\sigma_{m}$, required to accommodate counties with very noisy data.

### 4.3 Algorithmic results

**Fig 9:**
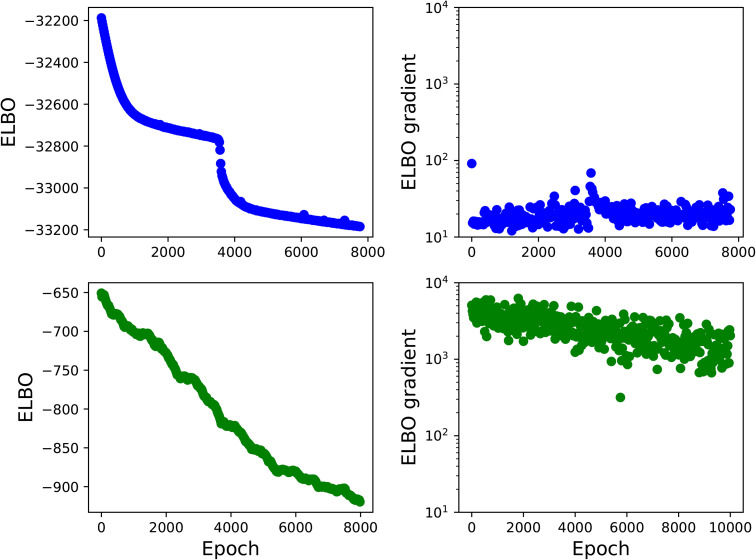
(Top) Convergence of the ELBO for the 33-county inversion along with the norm of the ELBO gradient as a function of gradient descent iterations for the reparametrized gradient formulation of MFVI. (Bottom) Convergence of the ELBO for a 1-county inverse problem along with the norm of the ELBO gradient for the black box formulation of MFVI. In both cases, $N_{s}=300$ samples were used for the MC estimators of the gradient.

Finally, we investigate the numerical aspects of the reparametrized algorithm described in § 3.2.1. The convergence of MFVI is depicted in Fig 9 where the ELBO and the norm of its gradient are shown as a function of gradient descent iterations.

The top of Fig 9 shows the ELBO and gradient for the 33-county calibration using the reparametrization formulation while the bottom provides a comparison to using black box VI for 1-county calibration of Bernalillo. In both cases, $N_{s}=300$ samples were used for the reparametrization and score function MC estimators of the gradient. Note that even for a single county, black box VI shows significantly higher variance in the gradient leading to poor convergence in comparison to the much larger 33-county problem calibrated with reparametrization. The convergence of the ELBO in the top row is also quite smooth suggesting that significantly less samples could be used to obtain good estimates of the gradient with the reparametrization approach. Hence, it is clear that reparametrization is necessary to scale the calibration to the 33-county inversion despite the added complexity of obtaining gradients of the log-likelihood. Note that the ELBO shows a sudden drop-off in Fig 9. High-dimensional, nonlinear objective functions tend to be non-convex and such sharp drop offs are common. This is not seen as often with lower-dimensional problems which explains why we see it only in the 33-county inversion.

**Fig 10:**
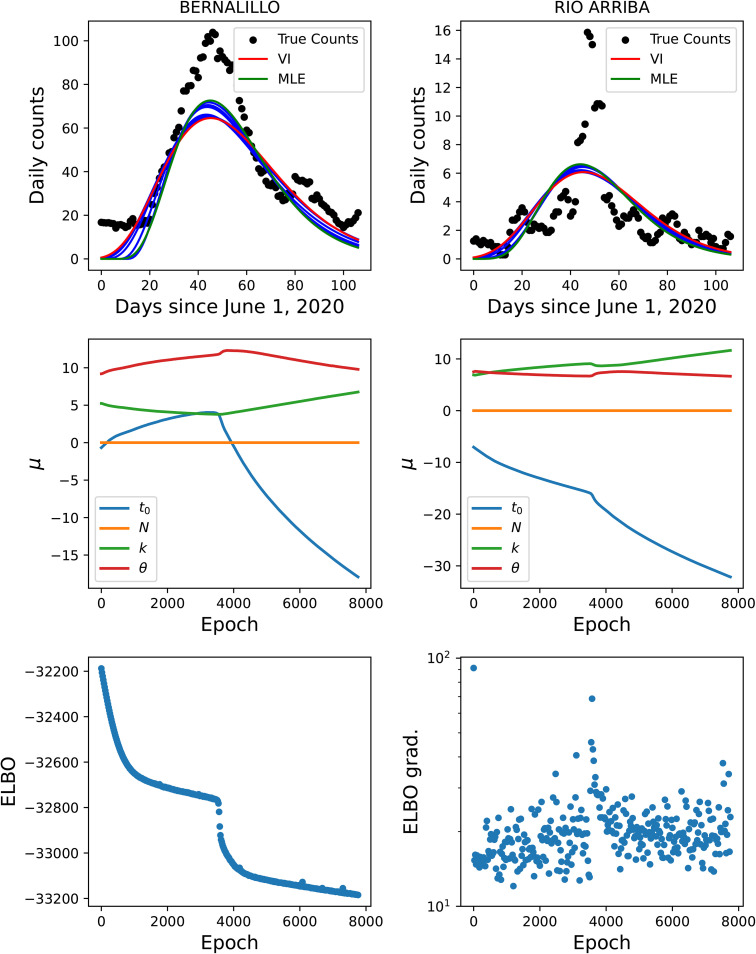
(Top) Convergence information corresponding to 33-county inversion using MFVI. Results corresponind to Bernalillo (left) and Rio Arriba (right). (Top) The MFVI mean initial condition is the MLE solution shown in red. Intermediate solutions are shown in blue and the final mean solution in green. (Middle) Corresponding convergence of the model parameters. (Bottom) The ELBO objective (left) and its gradient (right) as a function of iteration for the full 33 county inversion.

The top two rows of Fig 10 display convergence information from two counties, Bernalillo and Rio Arriba, taken from the 33-county inversion. Bernalillo and Rio Arriba were chosen as they have larger and smaller populations, respectively. The mean of the initial condition for MFVI is given by a MLE solution shown in red. Intermediate solutions are shown in blue along with the final solution in green. Observe that while similar to the MLE, MFVI subtly expands the shape of the wave to better cover the tail of the outbreak. This is potentially an effect of the tendency of the KL-divergence to increase the overlap of the surrogate and true posterior distributions at some expense of the mean prediction fitting the data less accurately. Comparing Figs 3–10, we can see that the coupled, 33-county inversion introduces a bias in the parameter estimates for Bernalillo. This is due to the multitude of NM counties that are sparsely populated and display significant noise in their daily case counts. Despite this effect, the outbreak detection performs well on the 33-county inversion data suggesting that the predictive uncertainties provided by the calibration remain informative.

*To summarize*, reparametrization provided us with the scalability needed to solve the high-dimensional inverse problem conditioned on data from all 33 NM counties.

## 5 Discussion

The results in § 4 show that MFVI can estimate an infection-rate field and the posterior distribution can be used to produce PPT runs. The MFVI parameter estimates do not quite agree with the AMCMC estimates [8,9], but their effect on the PPT runs is muted, as seen in the comparison in Fig 4 and the CRPS summaries in Table 1. Given that the inversion is a smoothing operation, i.e., we learn the infection-rate from historical data, any forecast produced with the estimated infection-rate will be predictive only if the epidemiological dynamics do not change. Consequently, if the forecast and data disagree, it could indicate the arrival of a new wave of infection. Since § 4 showed that MFVI and MCMC PPT results were similar, we henceforth use MFVI PPT runs to implement our outbreak detector. There does not seem to be a way to detect the discrepancy between MFVI and AMCMC posteriors without computing both.

### 5.1 Temporal detection

**Fig 11:**
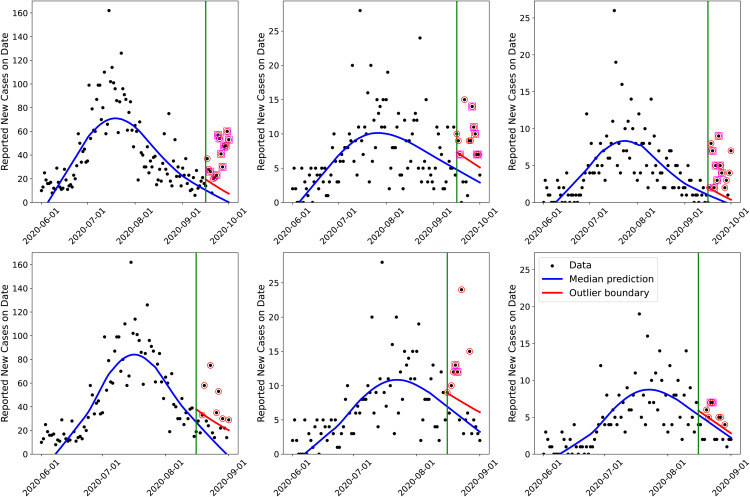
The infection-rate detector implemented using the infection-rate from the joint MFVI estimation of three counties (corresponding to Fig 4 for Bernalillo (left column), Santa Fe (middle column), and Valencia (right column). The symbols are the case-counts, the solid blue line the median forecast with the infection-rate, post-calibration and the solid red line is the alarm boundary. Outliers are circled and an alarm, corresponding to three consecutive outliers, is encased in a square. The vertical line is the point beyond which we forecast and thus test for the arrival of the Fall 2020 wave. Top row: Testing for arrival after September 15^th^, 2020. Bottom row: Testing for arrival after August 15^th^, 2020.

The argument above is used to fashion an outbreak detector using PPT runs. The detector works as follows. We sample the posterior in the same manner as for PPT runs, and use ***Y*** to compute an “outlier boundary”; we define it as the $99^{th}$ percentile of the forecasts. ***Y*** is computed using data from June 1^st^ to an end date (usually August 15^th^ or September 15^th^) where we test for a change in the epidemiological dynamics. The actual test consists of comparing a two-week-ahead forecast with the data that was observed during that period. Any day with case-counts above the “outlier boundary” is deemed an outlier. Three consecutive outlier days cause an “alarm”, corresponding to an anomalous change in the disease dynamics. Using this detector, when solving the problem with AMCMC [8,9], we found that we could detect the arrival of the Fall 2020 wave correctly, when tested using data up to September 15^th^. In Fig 11 we repeat the same test, but the infection-rate is estimated using MFVI. The top row depicts the outbreak detector being applied beyond September 15^th^, 2020. We see outliers and alarms for all three counties with a week of September 15^th^, i.e., the Fall 2020 wave was easily detected within a week’s worth of data. The bottom row repeats the test, but applied to August 15^th^. We see that Santa Fe and Valencia incur false positives, whereas Bernalillo does not. This implies that the approximations in MFVI may lead to erroneous detections for borderline cases (i.e., counties with small-count data and high variance noise) but areal units with large case-counts might be unaffected. The reason for the false positives is simple – the data is very noisy and the outbreak detector makes no attempt to reduce the variance in the noise. Comparing the correct detection on the top row of Fig 11 with the false positives in the bottom row, we see that outliers and alarms are plentiful after September 15^th^ and sporadic after August 15^th^. A slightly more sophisticated detector that performed temporal averaging could eliminate such false positives. We will address this below, using a simple spatio-temporal method.

**Fig 12:**
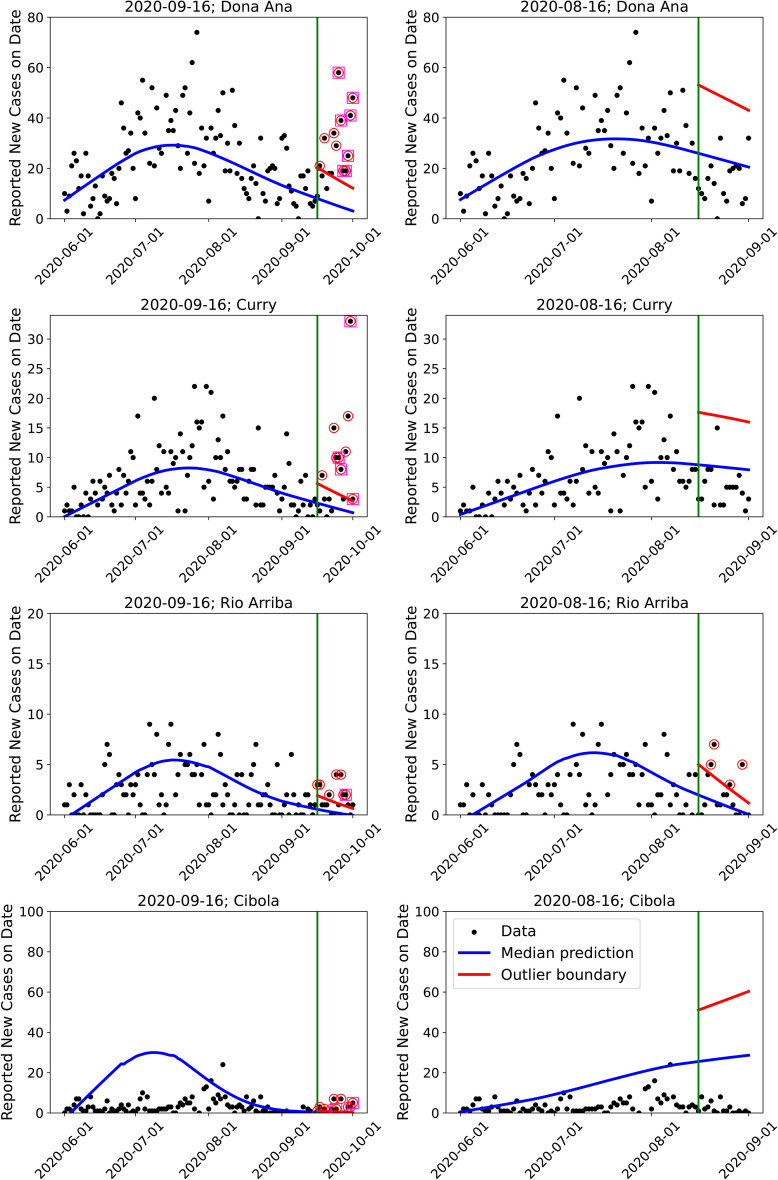
Performance of the outbreak detector for a “good” (Doña Ana), two “middling” (Curry and Rio Arriba) and one “bad” county (Cibola), as determined using CRPS in Fig 6. Left column: Testing for Fall 2020 wave arrival on September 15^th^, 2020. Right column: The same, but detection performed on August 15^th^. We do not see any false positives in the right column.

In Fig 12, we demonstrate the outbreak detector on a “good” (Doña Ana), two “middling” (Curry and Rio Arriba) and one “bad” county (Cibola), as determined using CRPS in Fig 6. The plotting conventions are the same as in Fig 11. We see that we correctly detect the Fall 2020 wave when testing on September 15^th^ and do not incur any false positive on August 15^th^. This performance is rather fortuitous for Rio Arriba and Cibola, which have unexplained case-count peaks in mid-July (Rio Arriba) and in late July (Cibola). It would be difficult to estimate an infection-rate profile for either of these counties independently (this is especially true for Cibola, where the *reported* case-counts show no wave-like structure); however, our spatial GMRF model regularizes the inversion and constructs an infection-rate stably. This also shows the necessity of smoothing in time guided by a process immune to flaws in the data – in our case, this is achieved via the incubation period distribution (*F*_*inc*_) and the infection-rate profile *f*_*inf*_ (see Eq. 1). (This smoothing would be far more difficult with a purely data-driven approach such as a pCAR-ST, which would attempt to accommodate the July peaks in the two counties.) Forecasts using this infection-rate do not match the case-count data at all, as is clear in the plots for Cibola, but provides us with stable PPT results and a perhaps meaningless detection. However, numerical and algorithmic stability in the face of poor quality data encourage us to believe that the MFVI algorithm and outbreak detection can be automated.

### 5.2 Detecting spatial patterns

The results above show that the MFVI estimates of the infection-rate have the ability to detect the Fall 2020 wave, especially for the counties in the first quartile (plotted in Fig 6), though our crude detector, which does not smooth / de-noise the data, might suffer from false positives due to high variance noise in observed case-count. However, the MFVI infection-rate *field* allows prediction in space and time, which allows spatio-temporal assimilation of data. We address this next.

**Fig 13:**
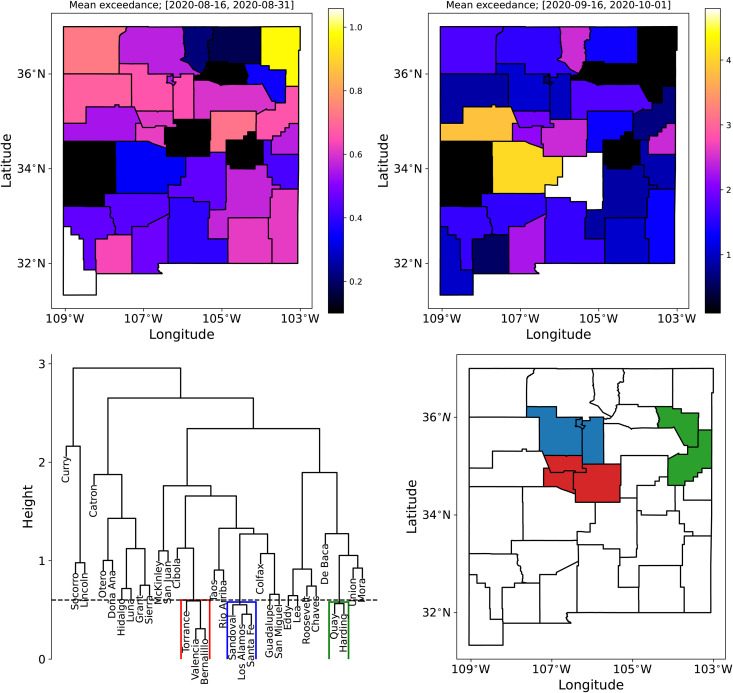
Top: Plot of mean exceedance $\overset{―}{\gamma_{r}}$ for NM, where $\overset{―}{\gamma_{r}}$ is averaged over August 15^th^ and August 31^st^ (left) before the arrival of the Fall 2020 wave and September 16^th^ and October 1^st^ (right), when the Fall 2020 wave had arrived. Bottom left: Dendrogram from hierarchical clustering, with the cut at a height of 0.6, resulting in clustering of counties. Bottom right: Disease clusters from the dendrogram.

Define “exceedance” $\gamma_{r,i}=y_{r,i}^{obs}/y_{r,i}^{\left(99\right)}$ for region *r* and day *i*. Here $y_{r,i}^{obs}$ is the observed case-counts and $y_{r,i}^{\left(99\right)}$ is the alarm boundary (the 99^th^ percentile computed from ***Y***). $\gamma_{r,i}>1$ would denote an outlier. Let


$$\begin{array}{c} \overset{―}{\gamma_{r}}=\frac{1}{N_{smooth}}\sum_{i=1}^{N_{smooth}}\gamma_{r,i} \end{array}$$

where *N*_*smooth*_ is a time-period over which we will average out the temporal noise and detect spatial patterns. Fig 13 (top row) shows a map of $\overset{―}{\gamma_{r}}$ computed over a two-week period after August $15^{th}$ (on the left) and a two-week period after September $15^{th}$ (on the right) when the Fall 2020 wave had arrived. We see from the colormap that the $\overset{―}{\gamma_{r}}\leq 1$ before the arrival in all counties except one; specifically, the false positives seen in Fig 11 for Santa Fe and Valencia are no longer visible. After the arrival, we see clear spatial structures on the right. Thus simply averaging the data in time provides a more robust detection, though with a loss of timeliness. However, we see two adjacent counties (in shades of yellow) with very high $\overset{―}{\gamma_{r}}$.

Epidemiological activity spreads due to population mixing and is largely between adjoining NM counties because of their large spatial expanse. Therefore adjacent counties are expected to have similar epidemiological behavior. If the epidemiological activity is represented using a derived / inferred / estimated quantity, and adjoining areal units differ greatly, it is either a consequence of erroneous estimation or an artifact of noise in data, i.e., spatial auto-correlation might be a simple mechanism for removing erratic behavior in data. To this end, we subject the $\overset{―}{\gamma_{r}}$ map in Fig 13 (top right) to clustering. The centroids of the counties serve as the spatial feature whereas $\overset{―}{\gamma_{r}}$ serves as a measure of how strong the Fall 2020 wave is, in each county. The data was Z-scored and subjected to hierarchical clustering using the R Statistical Software [55] (R version 4.3.2 (2023-10-31)) package stats, specifically the function hclust(). The resulting dendrogram is in Fig 13 (bottom left), which is cut at a height of 0.6 (corresponding to a quantile of 0.15) to reveal 3 clusters of counties. These are plotted in Fig 13 (bottom right) and reveal the same clustering as was seen in Fig 13 (top right). However, the counties in yellow were eliminated – their level of $\overset{―}{\gamma_{r}}$, though high, were too different and thus violated the need for spatial auto-correlation. Two of the clusters surround the county of Bernalillo, the population center of NM, where COVID-19 was very active in the city of Albuquerque.

Temporal epidemiological anomaly detections and spatial clusterings can be forged into a rough-and-ready test for the credibility of an outbreak detection. Questions of detection credibility arise due to the quality of the data used in outbreak detection and the approximations inherent in MFVI, which lead to narrow posterior distributions. Diseases spread, and an areal unit which shows persistently high disease levels while surrounded by quiescent areal units, is difficult to explain. This unexplainable behavior may simply be an erroneous detection and thus could be ignored.

## 6 Conclusion

In this paper, we have developed a scalable but approximate method to estimate the infection-rate field of an outbreak, defined over a collection of areal units. The method was demonstrated using COVID-19 data from the counties of NM. The purpose of estimating the infection-rate field was to detect a sudden change in the epidemiological dynamics, corresponding to the arrival of a new wave of infections. Contemporary methods use case-counts to perform this detection and are plagued by stochasticity and reporting errors in the data, especially when case-counts are low (as may be expected from sparsely-populated regions); in contrast, the infection-rate is governed by human mixing patterns that do not vary erratically day-to-day. Our method is based on mean-field variational inference (MFVI), but required some innovations for computational efficiency and for enforcing non-negativity constraints in the variables being estimated. MFVI addresses the issue of spatial auto-correlation in the COVID-19 dataset with a Gaussian Markov Random field.

The MFVI method obtains its scalability (in terms of the number of variables being estimated) by imputing a parametrized form for the posterior density, a set of independent Gaussians in our case; thereafter we estimate their means and standard deviations using scalable gradient-based algorithms. We compared the predictive skill of disease models that used the infection-rate field estimated using AMCMC versus MFVI to answer the research questions posed in § 1. Our findings are listed below.

- We find that the predictive skills of AMCMC- and MFVI-calibrated epidemiological models are similar, even though the parameters’ posterior distributions estimated by the two method are somewhat different. The uncertainties in the parameters estimated from MFVI are too small to be credible, in line with previous findings [51]. Both the approaches also estimate the noise in the data (i.e., the component of data variability that cannot be represented by the disease model), and the MFVI estimate is far larger than the AMCMC counterpart. In this manner MFVI compensates for its spuriously low uncertainty estimates in the parameters of the disease model, and achieves similar levels of predictive skill as the AMCMC method (which makes no approximations).
- The GMRF spatial model plays an important role in stabilizing the inversion. It regularizes the estimation problem with a Gaussian Markov Random Field, and is sufficient to stabilize the estimation when the observed case-counts from certain areal units (counties) bear no resemblance to the waxing and waning of the COVID-19 pandemic that is clearly observed in case-count data aggregated to the state level. Counties like Cibola and Rio Arriba, where errors / shortcomings in the data do not allow a reliable estimation of the infection-rate are regularized by it, and in the case of Rio Arriba also provides a credible forecast of the epidemiological dynamics. This robustness to occasional low quality data, which is to be expected for large inversions, allows the automation of the estimation process without any manual “cleaning” of the data and imputations of “cleaned” data values.
- The infection-rate field, estimated using MFVI, is used to detect the arrival of the Fall 2020 wave of COVID-19 in NM. To do so, we design a crude temporal anomaly detector which contrasts model forecasts with observed data; large discrepancies imply a change in epidemiological dynamics from the past. We had no difficulty in detecting the wave when it was present, but suffered from false positives when tests were conducted using data from before the arrival. These false positives were caused by high-variance noise in low case-count data and could be removed by temporal averaging. However, by doing so, our detections were no longer very timely. In addition, the availability of an infection-rate *field* allowed us to exploit the spatial auto-correlation to remove counties with spuriously high levels of epidemiological activity when their neighbors were quiescent. It is clear that MFVI yields useful information about the outbreak signature and does so in a scalable fashion.

Note that we made no attempt to design a proper anomaly detector with tunable parameters to trade-off specificity versus sensitivity and plot Receiver Operation Characteristic curves; our aim was to merely test of the existence of certain information in the infection-rate estimates. However, the study revealed that a proper anomaly detector would either have to smooth out high-variance noise in low case-count data, or be formulated using Negative Binomial or Poisson assumptions; these will be necessary to suppress false positives when the data has high-variance noise. False negatives, on the other hand, may not require any special considerations; this is certainly true for the NM COVID-19 dataset.

Note that other, non-Gaussian distributions may result in a more accurate posterior approximation through variational inference. In general, any distribution with finite moments could theoretically be used but may result in a more complex variational inference algorithms depending on the tractability of its statistics. Exploration of the optimal distribution assumption is left for future work.

A software implementation of the method described in this paper and the associated data can be found at our GitHub repository [61]. The specific version of the software used in this paper, and the necessary data, can also be found in Figshare [62].

## Appendix

### A Variational Inference

#### A.1 Score gradients of the ELBO.

We briefly review the score estimator, or black-box, approach to estimating the gradient of the ELBO. Recall that the ELBO is given by 9 which, for the sake of clarity, can be written in a more generic form


$$\begin{array}{c} ℒ(\phi )={𝔼}_{q(\theta ;\phi )}\left[f(\theta )\right] \end{array}$$ (14)

where f(θ) encapsulates the dependence on the random vector θ. To derive an estimator for the gradient, we can carry out the following manipulations


$$\begin{array}{c} \nabla_{\phi }ℒ(\phi )=\nabla_{\phi }{𝔼}_{q(\theta ;\phi )}\left[f(\theta )\right] \end{array}$$ (15)

$$\begin{array}{c} =\nabla_{\phi }\int q(\theta ;\phi )f(\theta )d\;\theta \end{array}$$ (16)

$$\begin{array}{c} =\int \nabla_{\phi }q(\theta ;\phi )f(\theta )d\;\theta \end{array}$$ (17)

$$\begin{array}{c} =\int q(\theta ;\phi )\frac{\nabla_{\phi }q(\theta ;\phi )}{q(\theta ;\phi )}f(\theta )d\;\theta \end{array}$$ (18)

$$\begin{array}{c} ={𝔼}_{q(\theta ;\phi )}\left[f(\theta )\nabla_{\phi }\log q(\theta ;\phi )\right] \end{array}$$ (19)

Hence, the gradient can be expressed as an expectation with respect to q(θ;ϕ) where only the log of the surrogate posterior needs to be differentiated with respect to the variational parameters ϕ.

#### A.2 Reparametrization gradients of the ELBO.

The likelihood and log likelihood are given by


$$\begin{array}{c} p(𝒟|\theta )=\prod_{i=1}^{N_{d}}2\pi^{-N_{r}/2}\det(\Sigma_{i}{)}^{-1/2}\exp\left(-\frac{1}{2}({𝐲}_{i}^{\left(o\right)}-{𝐲}_{i}{)}^{T}\Sigma_{i}^{-1}({𝐲}_{i}^{\left(o\right)}-{𝐲}_{i})\right) \end{array}$$ (20)

$$\begin{array}{c} l(\theta )=-\frac{N_{d}N_{r}2\pi }{2}-\frac{1}{2}\sum_{i=1}^{N_{d}}\log\det(\Sigma_{i})+({𝐲}_{i}^{\left(o\right)}-{𝐲}_{i}{)}^{T}\Sigma_{i}^{-1}({𝐲}_{i}^{\left(o\right)}-{𝐲}_{i}) \end{array}$$ (21)

Using the reparametrization trick, we can write the ELBO (9) and its gradient in the form


$$\begin{array}{c} ℒ(\phi )=-ℍ[q(\theta ;\phi )]-{𝔼}_{q(\epsilon )}[\log p(𝒟|\theta (\epsilon ,\phi ))+\log p(\theta (\epsilon ,\phi ))] \end{array}$$ (22)

$$\begin{array}{c} \nabla_{\phi }ℒ(\phi )=-\nabla_{\phi }ℍ[q(\theta ;\phi )]-{𝔼}_{q(\epsilon )}[\nabla_{\phi }\log p(𝒟|\theta (\epsilon ,\phi ))+\nabla_{\phi }\log p(\theta (\epsilon ,\phi ))] \end{array}$$ (23)

where ϕ=(μ,ρ), θ=μ+σ(ρ)⊙ϵ with ϵ~𝒩(0,𝐈). Here, σ is a positive transformation of the unconstrained variable ρ to ensure the variance is constrained to be positive. A Monte Carlo estimator of the gradient can then be written as


$$\begin{array}{c} \nabla_{\phi }ℒ(\phi )\approx -\nabla_{\phi }ℍ[q(\theta ;\phi )]-\frac{1}{N_{s}}\sum_{i=1}^{N_{s}}\nabla_{\phi }\log p(𝒟|\theta (\epsilon_{i},\phi ))+\nabla_{\phi }\log p(\theta (\epsilon_{i},\phi )) \end{array}$$ (24)

$$\begin{array}{c} =-\nabla_{\phi }ℍ[q(\theta ;\phi )] \end{array}$$ (25)

$$\begin{array}{c} -\frac{1}{N_{s}}\sum_{i=1}^{N_{s}}\left(\nabla_{\theta }\log p(𝒟|\theta (\epsilon_{i},\phi ))+\nabla_{\theta }\log p(\theta (\epsilon_{i},\phi ))\right)⊙\nabla_{\phi }\theta (\epsilon_{i},\phi ) \end{array}$$ (26)

where the last line is given by the chain rule and the fact that θ is defined by an element-wise transformation of ϕ. Observe that


$$\begin{array}{c} \nabla_{\mu }\theta =1 \end{array}$$ (27)

$$\begin{array}{c} \nabla_{\rho }\theta =\nabla_{\rho }\sigma (\rho )⊙\epsilon_{i} \end{array}$$ (28)

$$\begin{array}{c} \nabla_{\mu }ℍ[q(\theta ;\phi )]=0 \end{array}$$ (29)

$$\begin{array}{c} \nabla_{\rho }ℍ[q(\theta ;\phi )]=\frac{1}{\sigma (\rho )}⊙\nabla_{\rho }\sigma (\rho ) \end{array}$$ (30)

so that it remains to compute the gradients of the log-likelihood and prior.

#### A.3 Gradients of the log likelihood.

As the log likelihood factors independently across the data $i=1,\ldots ,N_{d}$, it suffices to compute the gradients $\nabla_{\theta }\log\det\Sigma_{i}$ and $\nabla_{\theta }({𝐲}_{i}^{\left(o\right)}-{𝐲}_{i}{)}^{T}\Sigma_{i}^{-1}({𝐲}_{i}^{\left(o\right)}-{𝐲}_{i})$ for a particular day *i*. The differentials of the these two terms are computed using matrix calculus [64] as


$$\begin{array}{c} \partial \log\det\Sigma_{i}=\text{Tr}(\Sigma_{i}^{-1}\partial \Sigma_{i}) \end{array}$$ (31)

$$\begin{array}{c} \partial ({𝐲}_{i}^{\left(o\right)}-{𝐲}_{i}{)}^{T}\Sigma_{i}^{-1}({𝐲}_{i}^{\left(o\right)}-{𝐲}_{i})=-({𝐲}_{i}^{\left(o\right)}-{𝐲}_{i}{)}^{T}\Sigma_{i}^{-1}(\partial \Sigma_{i})\Sigma_{i}^{-1}({𝐲}_{i}^{\left(o\right)}-{𝐲}_{i}) \end{array}$$ (32)

$$\begin{array}{c} -2({𝐲}_{i}^{\left(o\right)}-{𝐲}_{i}{)}^{T}\Sigma_{i}^{-1}\partial {𝐲}_{i} \end{array}$$ (33)

where the differential of $\Sigma_{i}$ is


$$\begin{array}{c} \partial \Sigma_{i}=(\partial \tau_{\Phi })[𝐃-\lambda_{\Phi }𝐖{]}^{-1}-(\partial \lambda_{\Phi })\tau_{\Phi }[𝐃-\lambda_{\Phi }𝐖{]}^{-1}𝐖[𝐃-\lambda_{\Phi }𝐖{]}^{-1} \end{array}$$ (34)

$$\begin{array}{c} +2\text{diag}(\sigma_{a}+\sigma_{m}{𝐲}_{i})[(\partial \sigma_{a})𝐈+(\partial \sigma_{m})\text{diag}({𝐲}_{i})+\sigma_{m}\text{diag}(\partial {𝐲}_{i})] \end{array}$$ (35)

We have the following derivatives:

**Case:** ${\hat{𝐦}}_{r}^{T}=({\hat{t}}_{0}^{r},{\hat{N}}^{r},{\hat{k}}^{r},{\hat{\theta }}^{r})$ are the unconstrained model variables for region *r* and ${\hat{\theta }}_{r}$ is a particular variable.


$$\begin{array}{c} \partial_{{\hat{\theta }}_{r}}{𝐲}_{i}=(0,\ldots ,\partial_{\theta_{r}}y_{r}(i;t_{0}^{r},N_{r},k^{r},\theta^{r})\theta_{i}^{\prime }({\hat{\theta }}_{i}),\ldots ,0) \end{array}$$ (36)

$$\begin{array}{c} \nabla_{{\hat{𝐦}}_{r}}\log\det\Sigma_{i}=2\sigma_{m}[\Sigma_{i}^{-1}{]}_{ii}\nabla_{{\hat{𝐦}}_{r}}y_{r}(i;t_{0}^{r},N^{r},k^{r},\theta^{r}) \end{array}$$ (37)

$$\begin{array}{c} \partial_{{\hat{\theta }}_{r}}({𝐲}_{i}^{\left(o\right)}-{𝐲}_{i}{)}^{T}\Sigma_{i}^{-1}({𝐲}_{i}^{\left(o\right)}-{𝐲}_{i})=-2\sigma_{m}({𝐲}_{i}^{\left(o\right)}-{𝐲}_{i}{)}^{T}\Sigma_{i}^{-1}\text{diag}(\sigma_{a}+\sigma_{m}{𝐲}_{i}) \end{array}$$ (38)

$$\begin{array}{c} \times \text{diag}(\partial_{{\hat{\theta }}_{r}}{𝐲}_{i})\Sigma_{i}^{-1}({𝐲}_{i}^{\left(o\right)}-{𝐲}_{i}) \end{array}$$ (39)

$$\begin{array}{c} -2({𝐲}_{i}^{\left(o\right)}-{𝐲}_{i}{)}^{T}\Sigma_{i}^{-1}\partial_{{\hat{\theta }}_{r}}{𝐲}_{i} \end{array}$$ (40)

**Case:** ${\hat{\theta }}_{r}={\hat{\tau }}_{\Phi }$


$$\begin{array}{c} \partial_{{\hat{\theta }}_{r}}{𝐲}_{i}=0 \end{array}$$ (41)

$$\begin{array}{c} \partial_{{\hat{\theta }}_{r}}\log\det\Sigma_{i}=\tau_{\Phi }^{\prime }({\hat{\tau }}_{\Phi })\text{Tr}(\Sigma_{i}^{-1}[𝐈-\lambda_{\Phi }𝐖{]}^{-1}) \end{array}$$ (42)

$$\begin{array}{c} \partial_{{\hat{\theta }}_{r}}({𝐲}_{i}^{\left(o\right)}-{𝐲}_{i}{)}^{T}\Sigma_{i}^{-1}({𝐲}_{i}^{\left(o\right)}-{𝐲}_{i})=\tau_{\Phi }^{\prime }({\hat{\tau }}_{\Phi })({𝐲}_{i}^{\left(o\right)}-{𝐲}_{i}{)}^{T}\Sigma_{i}^{-1} \end{array}$$ (43)

$$\begin{array}{c} \times [𝐈-\lambda_{\Phi }𝐖{]}^{-1}\Sigma_{i}^{-1}({𝐲}_{i}^{\left(o\right)}-{𝐲}_{i}) \end{array}$$ (44)

**Case:** ${\hat{\theta }}_{r}={\hat{\lambda }}_{\Phi }$


$$\begin{array}{c} \partial_{{\hat{\theta }}_{r}}{𝐲}_{i}=0 \end{array}$$ (45)

$$\begin{array}{c} \partial_{{\hat{\theta }}_{r}}\log\det\Sigma_{i}=\lambda_{\Phi }^{\prime }({\hat{\lambda }}_{\Phi })\tau_{\Phi }\text{Tr}(\Sigma_{i}^{-1}[𝐈-\lambda_{\Phi }𝐖{]}^{-1}𝐖[𝐈-\lambda_{\Phi }𝐖{]}^{-1}) \end{array}$$ (46)

$$\begin{array}{c} \partial_{{\hat{\theta }}_{r}}({𝐲}_{i}^{\left(o\right)}-{𝐲}_{i}{)}^{T}\Sigma_{i}^{-1}({𝐲}_{i}^{\left(o\right)}-{𝐲}_{i})=\lambda_{\Phi }^{\prime }({\hat{\lambda }}_{\Phi })\tau_{\Phi }({𝐲}_{i}^{\left(o\right)}-{𝐲}_{i}{)}^{T}\Sigma_{i}^{-1} \end{array}$$ (47)

$$\begin{array}{c} \times [𝐈-\lambda_{\Phi }𝐖{]}^{-1}𝐖[𝐈-\lambda_{\Phi }𝐖{]}^{-1}\Sigma_{i}^{-1}({𝐲}_{i}^{\left(o\right)}-{𝐲}_{i}) \end{array}$$ (48)

**Case:** ${\hat{\theta }}_{r}={\hat{\sigma }}_{a}$


$$\begin{array}{c} \partial_{{\hat{\theta }}_{r}}{𝐲}_{i}=0 \end{array}$$ (49)

$$\begin{array}{c} \partial_{{\hat{\theta }}_{r}}\log\det\Sigma_{i}=2\sigma_{a}^{\prime }({\hat{\sigma }}_{a})\text{Tr}(\Sigma_{i}^{-1}\text{diag}(\sigma_{a}+\sigma_{m}{𝐲}_{i})) \end{array}$$ (50)

$$\begin{array}{c} \partial_{{\hat{\theta }}_{r}}({𝐲}_{i}^{\left(o\right)}-{𝐲}_{i}{)}^{T}\Sigma_{i}^{-1}({𝐲}_{i}^{\left(o\right)}-{𝐲}_{i})=-2\sigma_{a}^{\prime }({\hat{\sigma }}_{a})({𝐲}_{i}^{\left(o\right)}-{𝐲}_{i}{)}^{T}\Sigma_{i}^{-1} \end{array}$$ (51)

$$\begin{array}{c} \times \text{diag}(\sigma_{a}+\sigma_{m}{𝐲}_{i})\Sigma_{i}^{-1}({𝐲}_{i}^{\left(o\right)}-{𝐲}_{i}) \end{array}$$ (52)

**Case:** ${\hat{\theta }}_{r}={\hat{\sigma }}_{m}$


$$\begin{array}{c} \partial_{{\hat{\theta }}_{r}}{𝐲}_{i}=0 \end{array}$$ (53)

$$\begin{array}{c} \partial_{{\hat{\theta }}_{r}}\log\det\Sigma_{i}=2\sigma_{m}^{\prime }({\hat{\sigma }}_{m})\text{Tr}(\Sigma_{i}^{-1}\text{diag}((\sigma_{a}+\sigma_{m}{𝐲}_{i})⊙{𝐲}_{i})) \end{array}$$ (54)

$$\begin{array}{c} \partial_{{\hat{\theta }}_{r}}({𝐲}_{i}^{\left(o\right)}-{𝐲}_{i}{)}^{T}\Sigma_{i}^{-1}({𝐲}_{i}^{\left(o\right)}-{𝐲}_{i})=-2\sigma_{m}^{\prime }({\hat{\sigma }}_{m})({𝐲}_{i}^{\left(o\right)}-{𝐲}_{i}{)}^{T}\Sigma_{i}^{-1} \end{array}$$ (55)

$$\begin{array}{c} \times \text{diag}((\sigma_{a}+\sigma_{m}{𝐲}_{i})⊙{𝐲}_{i}))\Sigma_{i}^{-1}({𝐲}_{i}^{\left(o\right)}-{𝐲}_{i}) \end{array}$$ (56)

The variable transformations are listed in Table 2.

**Table 2:** Variable transformations. s(x):log(exp(x)+1) is the *softplus* function and satisfies s(x)∈[0,1].

Variable	Constraint	Transformation
$t_{0}^{r}$	None	$id({\hat{t}}_{0}^{r})$
*N* ^*r*^	*N*^*r*^ ≥ 0	$\exp({\hat{N}}^{r})$
*k* ^*r*^	*k*^*r*^ ≥ 2	$s({\hat{k}}^{r})+2$
$\theta^{r}$	$k^{r}\geq \epsilon$	$s({\hat{\theta }}^{r})+\epsilon$
$\tau_{\Phi }$	$\tau_{\Phi }\geq 0$	$\exp({\hat{\tau }}_{\Phi })$
$\lambda_{\Phi }$	$0\leq \lambda_{\Phi }\leq 1$	$(1+\exp(-{\hat{\lambda }}_{\Phi }){)}^{-1}$
$\sigma_{a}$	$\sigma_{a}\geq 0$	$\exp({\hat{\sigma }}_{a})$
$\sigma_{m}$	$\sigma_{m}\geq 0$	$\exp({\hat{\sigma }}_{m})$

#### A.4 Approximation of model predictions and gradients via quadrature..

The model predictions **y**_*i*_, given by (2), involve a convolution integral that cannot be expressed in closed form. Hence, we approximate the predictions by integral quadrature


$$\begin{array}{c} y_{r}(i;t_{0}^{r},N^{r},k^{r},\theta^{r}) \end{array}$$ (57)

$$\begin{array}{c} =\int_{t_{0}^{r}}^{t_{i}}N_{r}f_{inf}(\tau -t_{0}^{r};k^{r},\theta^{r})(F_{inc}(t_{i}-\tau ;\mu ,\sigma )-(F_{inc}(t_{i-1}-\tau ;\mu ,\sigma ))d\;\tau \end{array}$$ (58)

$$\begin{array}{c} \approx \sum_{j=1}^{n}N_{r}w_{j}f_{inf}(\tau_{j}-t_{0}^{r};k^{r},\theta^{r})(F_{inc}(t_{i}-\tau_{j};\mu ,\sigma )-(F_{inc}(t_{i-1}-\tau_{j};\mu ,\sigma )) \end{array}$$ (59)

where *w*_*j*_, $\tau_{j}$ are quadrature weights and points given by a method such as Gaussian quadrature. As the function $\left(F_{inc}(t_{i}-\tau ;\mu ,\sigma )-(F_{inc}(t_{i-1}-\tau ;\mu ,\sigma )\right)$ does not depend on parameters θ, we can write it as ${\tilde{F}}_{inc}(\tau )$ for simplicity of notation. By the Leibniz integral rule, we can write the derivatives of the model predictions as


$$\begin{array}{c} \partial_{{\hat{\theta }}_{r}}{𝐲}_{i}=\int_{t_{0}^{r}}^{t_{i}}[\partial_{{\hat{\theta }}_{r}}N_{r}f_{inf}(\tau -t_{0}^{r};k^{r},\theta^{r})]{\tilde{F}}_{inc}(\tau )d\;\tau \;\text{if }{\hat{\theta }}_{r}\neq t_{0}^{r} \end{array}$$ (60)

$$\begin{array}{c} \partial_{{\hat{\theta }}_{r}}{𝐲}_{i}=\int_{t_{0}^{r}}^{t_{i}}[\partial_{{\hat{\theta }}_{r}}N_{r}f_{inf}(\tau -t_{0}^{r};k^{r},\theta^{r})]{\tilde{F}}_{inc}(\tau )d\;\tau -f_{inf}(0;k^{r},\theta^{r})\;\text{if }{\hat{\theta }}_{r}=t_{0}^{r} \end{array}$$ (61)

This requires that the functions $N_{r}f_{inf}(\tau -t_{0}^{r};k^{r},\theta^{r})]{\tilde{F}}_{inc}(\tau )$ and $\partial_{{\hat{\theta }}_{r}}N_{r}f_{inf}(\tau -t_{0}^{r};k^{r},\theta^{r})]{\tilde{F}}_{inc}(\tau )$ are continuous in τ and ${\hat{\theta }}_{r}$ in a region of the $\tau -{\hat{\theta }}_{r}$ plane including $t_{0}\leq \tau \leq t_{i}$, a condition that’s easily met by ensuring certain constraints are satisfied by the parameters $\left(t_{0}^{r},N^{r},k^{r},\theta^{r}\right)$ for $r=1,\ldots ,N_{r}$. These constraints are enforced via the variable transformations in (12). Hence, we can approximate (60) and (61) via quadrature in the form


$$\begin{array}{c} \partial_{{\hat{\theta }}_{r}}{𝐲}_{i}\approx \sum_{j=1}^{n}w_{j}[\partial_{{\hat{\theta }}_{r}}N_{r}f_{inf}(\tau_{j}-t_{0}^{r};k^{r},\theta^{r})]{\tilde{F}}_{inc}(\tau_{j})\;\text{if }{\hat{\theta }}_{r}\neq t_{0}^{r} \end{array}$$ (62)

$$\begin{array}{c} \partial_{{\hat{\theta }}_{r}}{𝐲}_{i}\approx \sum_{j=1}^{n}w_{j}[\partial_{{\hat{\theta }}_{r}}N_{r}f_{inf}(\tau_{j}-t_{0}^{r};k^{r},\theta^{r})]{\tilde{F}}_{inc}(\tau_{j})-f_{inf}(0;k^{r},\theta^{r})\;\text{if }{\hat{\theta }}_{r}=t_{0}^{r} \end{array}$$ (63)

Hence, in this implementation of VI, gradients of the ELBO have a pseudo-analytic form where “outer” gradients of the log likelihood with respect to model predictions are exact and “inner” gradients of the model predictions with respect to parameters are approximated via quadrature. This allows for accurate gradient approximations that can be calculated efficiently leading to a scalable VI algorithm that can be applied to the high-dimensional inverse problem for the outbreak model.

### B Infection-rate estimates

**Fig 14:**
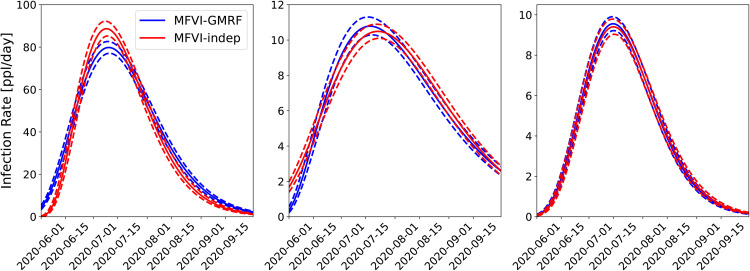
Comparison of the infection rate curves that determine predictions in Fig 3. These are for a 3-county inference of Bernalillo (left), Santa Fe (middle), and Valencia (right) done jointly using the GMRF model and independently for each county. The solid lines shows the median predictions and the dashed lines are the 5^th^ and 95^th^ percentiles.

4.1 describes the estimation of the infection-rate field over Bernalillo, Santa Fe and Valencia using MFVI jointly (where the GMRF spatial model is used) and individually. Fig 3 plots the PPT runs, driven by a distribution of infection-rate fields. The corresponding fields are in Fig 14.

**Fig 15:**
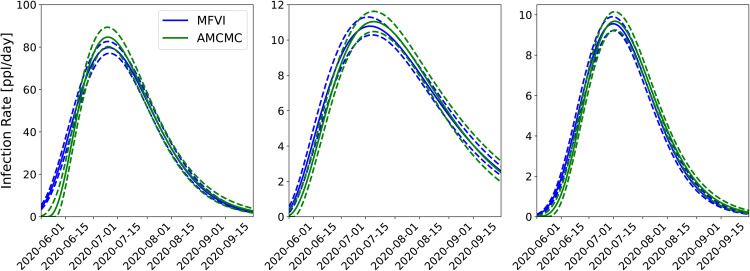
Comparison of the infection rate curves that determine predictions in Fig 4. These are for a 3-county inference of Bernalillo (left), Santa Fe (middle), and Valencia (right) done jointly using the MFVI and AMCMC (taken from Refs. [8,9]). The solid lines shows the median predictions and the dashed lines are the 5^th^ and 95^th^ percentiles.

The same section describes the estimation of the infection-rate field over Bernalillo, Santa Fe and Valencia using AMCMC and the MFVI. Fig 4 plots the PPT runs, driven by a distribution of infection-rate fields. The corresponding fields are in Fig 15.
